# Repurposing Copper(II)/THPTA as A Bioorthogonal Catalyst for Thiazolidine Bond Cleavage

**DOI:** 10.1002/advs.202408180

**Published:** 2024-09-19

**Authors:** Chengyun Ma, Guoqing Liu, Juan Yin, Jianan Sun, Disheng Luo, Dechun Yang, Shuo Pang, Wei Hou, Xinya Hemu, Bangce Ye, Xiaobao Bi

**Affiliations:** ^1^ Collaborative Innovation Center of Yangtze River Delta Region Green Pharmaceuticals, College of Pharmaceutical Sciences Zhejiang University of Technology Hangzhou 310014 China; ^2^ Zhejiang Yangshengtang Institute of Natural Medication Co., Ltd Hangzhou 310013 China; ^3^ School of Traditional Chinese Pharmacy China Pharmaceutical University Nanjing 210009 China; ^4^ Lab of Biosystem and Microanalysis, State Key Laboratory of Bioreactor Engineering East China University of Science and Technology Shanghai 200237 China

**Keywords:** bioorthogonal catalysts, bond cleavage reactions, copper ions, living systems, metals, thiazolidines

## Abstract

Metal‐mediated chemical transformations are promising approaches to manipulate and regulate proteins in fundamental biological research and therapeutic development. Nevertheless, unlike bond‐forming reactions, the exploration of selective bond cleavage reactions catalyzed by metals that are fully compatible with proteins and living systems remains relatively limited. Here, it is reported that Copper(II)/tris(3‐hydroxypropyltriazolylmethyl)amine (THPTA), commonly used in copper‐catalyzed azide‐alkyne cycloaddition (CuAAC) reaction, can be repurposed as a new bioorthogonal catalyst for thiazolidine (Thz) bond cleavage. This process liberates an α‐oxo‐aldehyde group under physiological conditions, without requiring additional additives. To showcase the utility of this method, this simple catalyst system is coupled with genetic code expansion technology to achieve on‐demand activation of genetically encoded Thz‐caged α‐oxo‐aldehydes, enabling further functionalization of proteins. For the first time, this cell‐compatible Thz uncaging reaction allows for the site‐specific installation of α‐oxo‐aldehydes at the internal positions of proteins in phage and bacterial surface display systems, expanding the chemical space of proteins. Overall, this study expands the toolkit of bioorthogonal catalysts and paves the way for metal‐promoted chemical reactions in living systems, potentially benefiting various applications in the future.

## Graphical abstract

Beyond click chemistry, this study unveils the previously unexplored capability of Copper(II)/tris(3‐hydroxypropyltriazolylmethyl)amine (THPTA) in thiazolidine (Thz) bond cleavage reactions under fully biocompatible conditions. This approach allows for the on‐demand liberation of α‐oxo‐aldehyde from one genetically encoded Thz ring, facilitating site‐specific modifications of proteins, phage particles, and living bacterial cell surfaces without compromising their normal functions.

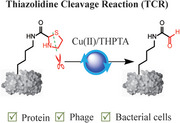

## 1 Introduction

Proteins play integral roles in almost all biological activities, sparking significant interest in their chemical modification and manipulation in basic biology studies and the development of protein therapeutics.^[^ ^1^ ^]^ One attractive approach in recent years is to use metal‐catalyzed reactions to endow proteins with novel properties or regulate their functions and activities both in vitro and in vivo.^[^ ^2^ ^]^ Particularly, metal‐triggered bond cleavage reactions are increasingly recognized as useful tools for on‐demand control of protein activity or prodrug release, owing to the versatility, tunability, better penetration and spatial targeting abilities of metals in living tissues and animals.^[^ ^3^ ^]^ Representative examples include Ruthenium (Ru)‐mediated deallyloxycarbonyl (allyl carbamate) reaction,^[^ ^4^ ^]^ Ru‐triggered olefin metathesis reaction,^[^ ^5^ ^]^ Palladium (Pd)‐triggered deallylation,^[^ ^6^ ^]^ Pd‐mediated thiazolidine cleavage,^[^ ^7^ ^]^ Copper(I)‐triggered dimethyl propargyl group deprotection,^[^ ^8^ ^]^ Copper(I)‐dependent oxidative cleavage reaction,^[^ ^9^ ^]^ Platinum‐triggered deprotection of N‐propargyls,^[^ ^10^ ^]^ and Gold‐mediated propargyl deprotection.^[^ ^11^ ^]^ Despite these advances, compared to the extensive repertoire of bond‐forming reactions,^[^ ^12^ ^]^ metal‐triggered bond cleavage reactions, especially those compatible with living systems, remain relatively underexplored. Therefore, there is an urgent need to enrich current toolboxes and expand their utility in biomedical research.

**Scheme 1:**
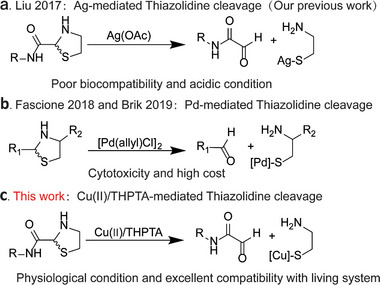
Metal‐mediated thiazolidine (Thz) cleavage reactions. a) Ag‐mediated reaction reported by Liu's group;^[^ ^13b^ ^]^ b) Pd‐mediated reaction reported by Brik and Fascione's group;^[^ ^7^, ^15^ ^]^ c) Cu(II)/THPTA‐catalyzed reaction developed in this work.

Thiazolidine (Thz) chemistry represents an interesting “click” type bioorthogonal reaction, involving the specific condensation of an aldehyde and 1,2‐aminothiol in aqueous buffers, and has been widely used for protein/peptide and living cell modifications.^[^ ^13^ ^]^ This chemistry is generally reversible under physiological conditions and also responsive to various stimuli including small molecules and transition metals.^[^ ^14^ ^]^ Thus, this unique property makes Thz chemistry a highly useful tool for temporarily masking or releasing aminothiols or aldehydes on demand. For instance, through chemical protein synthesis and genetic code expansion (GCE) technique, Brik, Fascione, and Liu's groups have introduced Thz linkages into protein backbones or side chains, independently developing Pd‐ and Silver (Ag)‐triggered Thz cleavage reactions to release the active aldehydes. This offers new strategies to regulate synthetic protein activity in living cells or to post‐functionalize recombinant proteins (**Scheme** **1**).^[^ ^7^, ^13^, ^15^ ^]^ Despite their utility in specific scenarios, these studies still suffer from inherent drawbacks such as being limited to protein‐level labeling, causing protein precipitation, poor water solubility of the catalyst, the requirement for other reducing additives or high cytotoxicity. Thus, a new bioorthogonal metal catalyst for Thz cleavage with significantly reduced cytotoxicity and excellent water solubility is still highly demanded. To address this gap, herein we screened commonly used metal ions and identified that Copper(II)/tris(3‐hydroxypropyltriazolylmethyl)amine (THPTA) can serve as a new bioorthogonal decaging catalyst system capable of cleaving Thz to release α‐oxo‐aldehydes under physiological conditions, without the need for additional additives. This simple catalyst system is also compatible with functional proteins, intact phage particles, and living bacterial cells. Moreover, phage and bacterial viability assays demonstrated that Cu(II)/THPTA exhibited much lower side‐effects and toxicity compared to previously used Pd ions. As a demonstration of the utility of this Thz decaging reagent, we generated site‐specifically labeled nanobody and phage particles, as well as dual‐labeled living bacterial cells, for cancer cell‐selective imaging and targeting.

## 2 Results and Discussion

### 2.1 Screening Metal Ions for Thz Cleavage on Proteins

**Figure 1:**
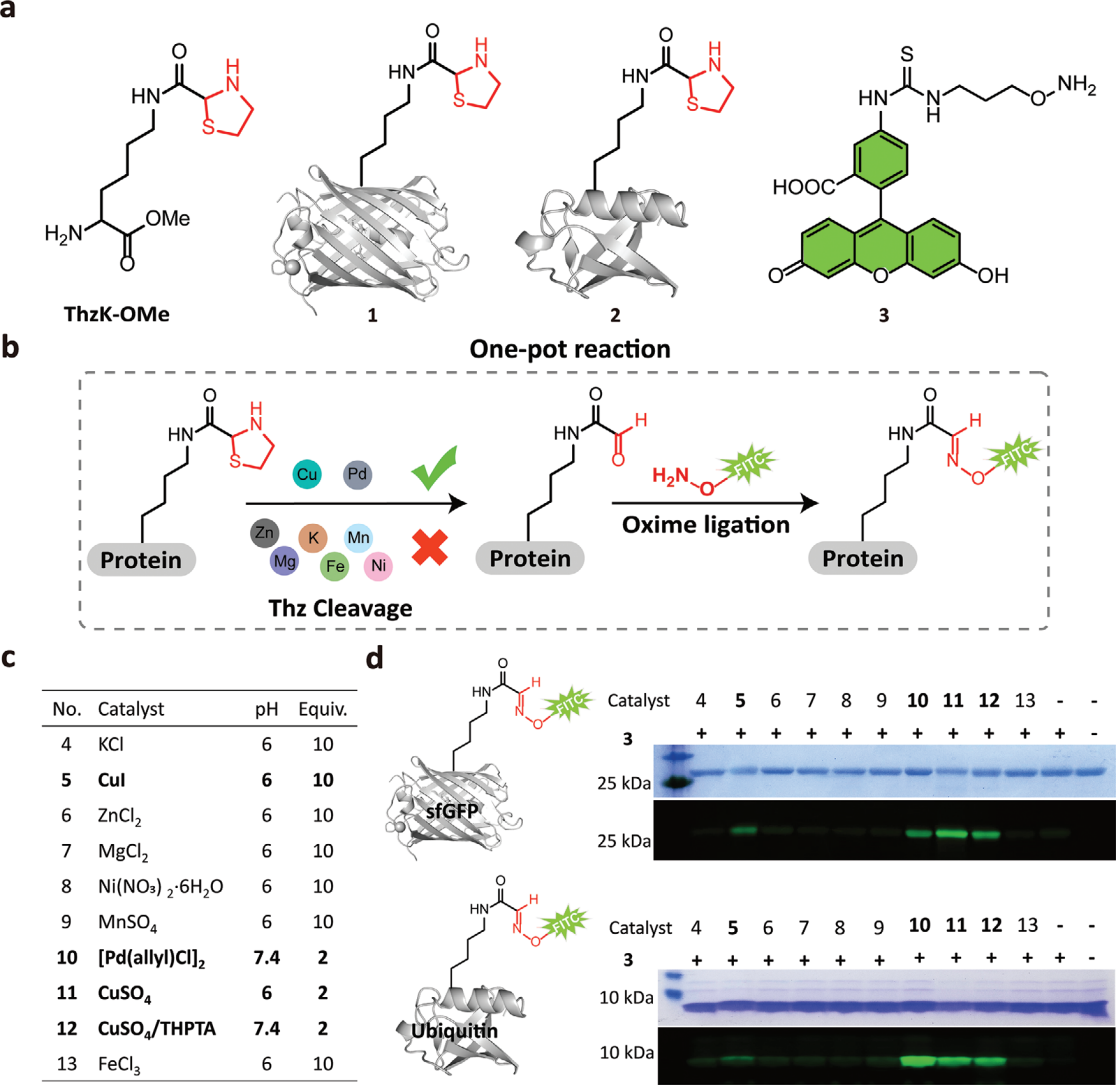
Screening of metal catalysts for thiazolidine deprotection. a) Chemical structures of ThzK‐OMe, model proteins incorporating ThzK‐OMe and the fluorescent probe used; b) General scheme for screening metal catalysts for Thz cleavage on proteins; c) Metal ions and reaction conditions used. Note: copper salt alone **5** and **11** exhibited poor solubility in Phosphate Buffered Saline (PBS), pH 7.4, so they were dissolved in a mildly acidic buffer (pH 6) in which it could be solubilized to some extent; d) SDS‐PAGE and fluorescent gel analysis of sfGFP‐ThzK **1** (top panel) and Ubi‐ThzK **2** (down panel) labeled with FITC probe **3** in the presence of different metal catalysts.

**Scheme 2:**
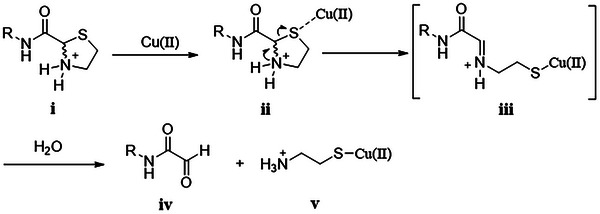
Proposed mechanism for Cu‐assisted thiazolidine bond cleavage.

For the purpose of catalyst screening, we generated two model proteins, sfGFP‐ThzK **1** and Ubiquitin (Ubi)‐ThzK **2**, by site‐specific incorporation of ThzK‐OMe using our established protocols (**Figure** **1**a; Scheme S1, Supporting Information).^[^ ^13b^ ^]^ To easily monitor the bond cleavage reaction, an aminooxy‐Fluorescein Isothiocyanate (FITC) probe **3** was prepared (Figure 1a) and co‐incubated with proteins **1** and **2** in the presence of the tested metal ions. Upon metal ion‐induced Thz cleavage, active α‐oxo‐aldehydes would be released, and the protein would then be labeled with FITC via oxime ligation. This allows us to monitor the reaction by detecting the fluorescent signal intensity on gels, thereby identifying which metal ions are involved in the Thz bond cleavage (Figure 1b). In our experimental setup, we selected several metal ions (**4**‐**13**) commonly used in biological assays and dissolved them within a physiologically relevant pH range (6‐7.4) (Figure 1c). Pd(II) **10** served as the positive control due to its known catalytic ability to break Thz bonds.^[^ ^15^ ^]^ Following the one‐pot reaction shown in Figure 1b, the results showed that both proteins **1** and **2** treated with Pd and Cu species exhibited apparent green fluorescent bands on the gels, indicating that the Thz ring was ruptured to release the aldehyde moiety. Interestingly, based on the intensity of the fluorescent bands, both free and coordinated copper ions can catalyze the Thz cleavage reaction, with Cu(II) ions **11** and **12** demonstrating greater efficiency than Cu(I) **5** (Figure 1d). Subsequent analyses showed that Cu(II)/THPTA complex was also more efficient than Cu(I)/THPTA in the reaction (Figure S1, Supporting Information). This finding is particularly intriguing. While copper ions and appropriate chelating ligands are well‐known for their use in copper‐catalyzed azide‐alkyne cycloaddition (CuAAC) reaction for various applications,^[^ ^16^ ^]^ there have been no reports of copper ions catalyzing Thz bond cleavage under physiological conditions. Therefore, we propose a mechanism in which copper ions in their free form or THPTA‐complexed form first bind to the sulfur atom of Thz (**i** and **ii**), leading to C‐S bond cleavage. This process produces intermediate **iii**, which then hydrolyzes to form aldehyde **iv** and cysteamine‐Cu(II) **v** in the presence of water (**Scheme** **2**). Overall, this study not only unveils a novel utility of copper ions in bioorthogonal cleavage reactions (BCRs) but also provides a potentially valuable method for labeling proteins and living cells under physiological conditions. In the following studies, Cu(II)/THPTA was chosen as the preferred catalyst for Thz bond cleavage due to its much lower cytotoxicity compared to Cu(I), as well as its good biocompatibility and excellent water solubility.^[^ ^17^ ^]^

### 2.2 Verification of Cu(II)/THPTA‐Triggered Thz Cleavage on Peptides

**Figure 2:**
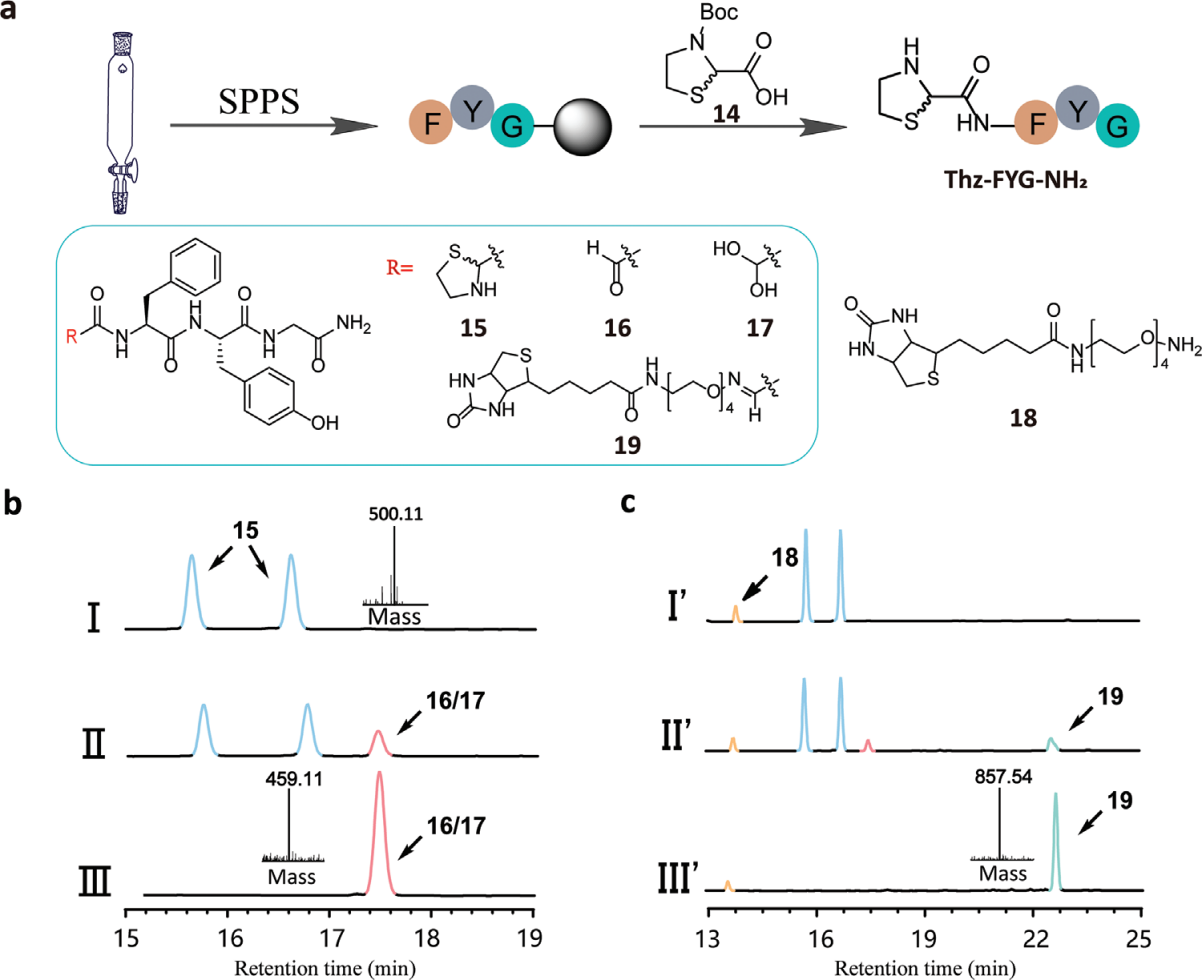
Modification of a short peptide via Thz cleavage and oxime ligation. a) Scheme for the generation of Thz‐containing peptide and chemical structures of peptides and reagents used. b) HPLC and MS analysis of peptide **15** and the Thz cleavage reaction. (I): the starting material **15** with two peaks; the observed mass is 500.11, calcd 499.19. (II) and (III): Thz cleavage reaction for 2 and 12 h, respectively; the light red peak represents the expected aldehyde **16** and hydrated form **17** with an observed mass of 459.11, calcd 458.18; c) HPLC and MS analysis of the one‐pot reaction containing **15**, Cu(II)/THPTA and **18** in PBS (pH 7.4) at 0, 1 and 12 h. (I’): 0 h, the aurantium peak is **18**. (II’): 1 h and (III’): 12 h, the cyan peak represents the conjugated product **19** with an observed mass of 857.54, calcd 856.90.

To further investigate the copper ion‐triggered Thz bond‐breaking reaction, we synthesized a short model peptide, Thz‐FYG‐NH_2_ **15** incorporating a Thz group at its N‐terminus using Fmoc chemistry via solid phase peptide synthesis (SPPS) (**Figure** **2**a). The purified peptide **15** was confirmed by both High Performance Liquid Chromatography (HPLC) and Mass Spectrometry (MS) analysis. Notably, due to the presence of mixed diastereomers in the Thz compound (3‐(*tert*‐butoxycarbonyl)thiazolidine‐2‐carboxylic acid) **14**, peptide **15** exhibited two peaks on the HPLC profile despite having identical mass values (Figure 2b, panel I). To assess whether Cu(II)/THPTA could catalyze the ring rupture reaction to generate the α‐oxo‐aldehyde, peptide **15** was treated with Cu(II)/THPTA (2 equiv.) in PBS (pH 7.4) at room temperature. Results showed that after 2 h, the expected α‐oxo‐aldehyde‐bearing peptide **16** and its hydrated form **17** were successfully detected, and after 12 h, **15** was quantitatively converted into the peptide aldehyde, as confirmed by ESI‐MS analysis (Figure 2b, panel II and III). To confirm the reactivity of the released α‐oxo‐aldehyde moiety, a one‐pot reaction was further conducted at room temperature by mixing peptide **15** (1 mM) with aminooxy‐biotin **18** (3 mM) and Cu(II)/THPTA (2 mM) in PBS (pH 7.4). An aliquot of the reaction mixture was analyzed by HPLC, revealing that the conjugated product **19** could be detected at 1 h, albeit with a low yield. However, extending the reaction time to 12 h resulted in quantitative conversion into the peptide conjugate **19**, as confirmed by ESI‐MS analysis (Figure 2c). Overall, this study validated the capability of Cu(II)/THPTA to cleave the Thz bond under physiological pH, releasing the active aldehyde for bioconjugation. This approach could be useful for peptide‐drug conjugate (PDC) development, as well as protein and living cell labeling studies.

### 2.3 Cu(II)/THPTA‐Triggered Thz Cleavage on 7D12 Nanobody

**Figure 3:**
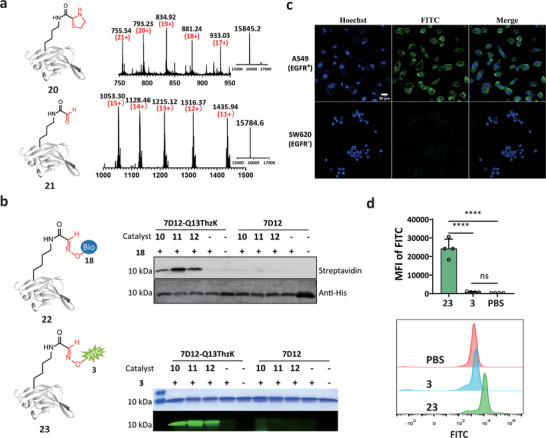
Generation of site‐specifically labeled 7D12 nanobody for cancer cell‐selective targeting. a) ESI‐MS analysis of 7D12‐Q13ThzK **20** with the observed mass 15845.2 Da, calcd 15845.4 Da (upper) and 7D12‐Q13‐CHO **21** with the observed mass 15784.6 Da, calcd 15786.3 Da (lower); b) Western blot and fluorescent gel analysis of chemically modified 7D12 with biotin and fluorescein. Compared to the wild type 7D12 group, biotin‐labeled 7D12 **22** (upper) and FITC‐labeled 7D12 **23** (lower) could only be detected in 7D12‐Q13ThzK treated by Pd(II) **10**, Cu(II) **11** and Cu(II)/THPTA **12**; WB: Streptavidin‐HRP conjugate and Anti‐His tag antibody were used; c) Confocal microscopy images of A549/SW620 cells incubated with 100 nM FITC‐labeled 7D12 **23** (green) for 30 min in 37 °C. The nuclei were stained with Hoechst (blue). Scale bars = 50 µm; d) Comparison of the mean fluorescence intensity of A549 cells treated with different samples using flow cytometry. One‐way ANOVA with Tukey´s multiple comparisons test: ns, not significant ^****^ *p* < 0.0001, n = 4.

To validate whether Cu(II)/THPTA‐mediated Thz cleavage is compatible with functional proteins and showcase its utility, we selected the nanobody 7D12 as a model. Previous studies have identified Gln13 as a permissive site for encoding unnatural amino acids (UAAs) without affecting its binding affinity to the epidermal growth factor receptor (EGFR),^[^ ^18^ ^]^ which is widely overexpressed on various cancer cells. So a pETDuet plasmid encoding the 7D12 nanobody with a Q13TAG mutation was constructed to express the protein incorporating ThzK‐OMe in the predertermined site via GCE technology. This plasmid was then co‐transformed into *Escherichia coli* (*E. coli*) BL21(DE3) with the pEVOL‐MbPylRS plasmid, which encodes the MbPylRS/tRNA pair that can specifically recognize ThzK‐OMe, to produce the 7D12‐Q13ThzK **20** in the presence of 2 mm ThzK‐OMe. It was shown that the full‐length of **20** could be obtained with ≈1 mg L^−1^ yield after immobilized metal affinity chromatography (IMAC) purification (Figure S2, Supporting Information). Subsequently, the integrity of **20** and its aldehyde derivative 7D12‐Q13‐CHO **21**, which was obtained after Cu(II)/THPTA treatment, was confirmed by ESI‐MS analysis (**Figure** **3**a). Following this, a one‐pot labeling reaction of **20** with probe **3** or **18** for 12–18 h was further conducted to verify whether it could be labeled or not. Western blot and fluorescent gel analysis showed that both biotinylated and fluorescent proteins (**22** and **23**) were detected only in the presence of Pd(II) **10**, Cu(II) **11**, Cu(II)/THPTA **12**, and ThzK‐OMe. In contrast, no signals were observed for wild type (WT) 7D12 lacking ThzK, regardless of the addition of the metal catalysts, or for 7D12‐Q13ThzK **20** in the absence of metal catalysts under identical conditions (Figure 3b). Similarly, treatment of sfGFP‐ThzK **1** and Ubi‐ThzK **2** under the same conditions yielded the same result, with expected biotinylated bands detected only in proteins treated with Pd(II) **10**, Cu(II) **11**, and Cu(II)/THPTA **12** (Figure S3, Supporting Information). This data validated the site‐selectivity and bioorthogonality of the Thz bond cleavage reaction. Next, to evaluate whether the FITC‐conjugated 7D12 **23** still retained its targeting EGFR ability after copper‐triggered Thz cleavage and oxime ligation, we tested its binding affinity toward two human cancer cell lines: lung cancer A549 (EGFR^+^) and colorectal cancer SW620 (EGFR^−^). Results showed that the distinct green rims were observed only in A549 cells, whereas no signal was detected in SW620 cells (Figure 3c). Flow cytometry analysis further supported these findings, showing a shift in the fluorescent intensity of A549 cells labeled with **23** compared to those treated by PBS and **3** only (Figure 3d). These data indicated the targeting capability and selectivity of labeled 7D12 toward EGFR‐positive cancer cells were well‐maintained, affirming that Cu(II)/THPTA treatment has no effect on the integrity of functional proteins and the method developed here has the potential for developing targeted protein molecules in diagnostic and therapeutic applications.

### 2.4 Cu(II)/THPTA‐Triggered Thz Cleavage on Phage Particles

**Figure 4:**
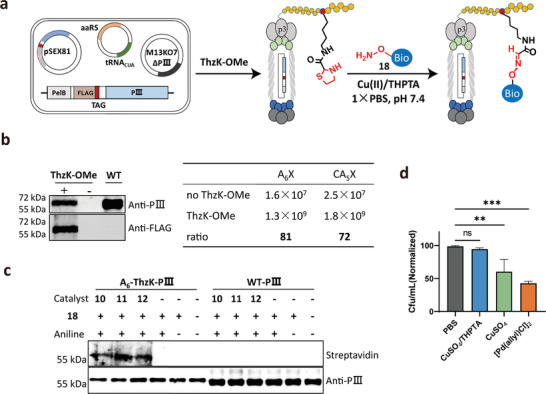
Site‐specific incorporation of α‐oxo‐aldehyde and modification of phage particles. a) Schematic diagram illustrating the site‐specific incorporation of ThzK‐OMe into the pIII proteins using a tri‐plasmid system, followed by their chemical modification using the aminooxy‐biotin probe **18** via a Cu(II)/THPTA‐mediated Thz cleavage reaction and oxime ligation; b) Western blot detection of ThzK‐OMe incorporation into phages. Only in the presence of ThzK‐OMe were both pIII protein and FLAG‐tag simultaneously detected in the FLAG‐A_6_X‐pIII phages using anti‐pIII and anti‐FLAG antibodies, indicating the expression of full‐length FLAG‐A_6_ThzK‐pIII. No FLAG band was detected in the WT phages (left panel); The comparison of phage yields (Cfu/mL) for two phage variants (one featuring an N‐terminal A_6_X peptide and the other with an N‐terminal CA_5_X peptide) in the presence and absence of ThzK‐OMe (right panel); c) Western blot analysis of phages labeled with probe **18**. In contrast to WT phages, biotinylated pIII bands were only detected in A_6_ThzK‐pIII phages treated with Pd(II) **10**, Cu(II) **11**, or Cu(II)/THPTA **12**; d) Comparison of the effect of metal ions on phage activity. Data are normalized to respective controls. One‐way ANOVA with Dunnett`s multiple comparisons test: ns, not significant ^**^ *p* < 0.01, ^***^ *p* < 0.001; n = 3.

Phages are naturally occurring nanoparticles that carry multiple chemical groups on their surfaces, making them attractive scaffolds in drug delivery, nanotechnology, and material sciences when combined with various chemical modification techniques. However, to date, there has been a lack of methods capable of site‐specifically incorporating α‐oxo‐aldehydes into the internal positions of peptides/proteins displayed on phages. Previous methods have limited α‐oxo‐aldehyde generation to the N‐terminus of phage proteins through serine oxidation and pyridoxal 5′‐phosphate (PLP)‐mediated transformation, and phage activity may also be compromised.^[^ ^19^ ^]^ Therefore, we aimed to investigate whether Cu(II)/THPTA‐triggered Thz cleavage could serve as a new and mild approach for the site‐specific incorporation of aldehydes into phages. To achieve this, we adopted a tri‐plasmid system for ThzK‐OMe incorporation into phages according to previous studies.^[^ ^20^ ^]^ In this system, the pSEX81 plasmid encoded a FLAG tag followed by A_6_X‐pIII or CA_5_X‐pIII fusion protein, where A_6_ and A_5_ denote the poly‐Ala sequence and X denotes the incorporated ThzK‐OMe. Meanwhile, a genetically engineered M17KO7 helper phage, with the deletion of pIII gene, was also introduced into the host bacterial cells. The deletion of the pIII in the M13KO7 helper phage rendered it unable to produce viable phages (Figure S4, Supporting Information). Only when ThzK‐OMe was successfully incorporated into the X position could the full‐length pIII (FLAG tag‐A_6_ThzK‐pIII or FLAG tag‐CA_5_ThzK‐pIII) fusion proteins be produced. This process ensures that phages with ThzK incorporation are produced for further functionalization, while the wild‐type phages lacking ThzK are not (**Figure** **4**a). Following this scheme, the full‐length expression of pIII incorporating ThzK was subsequently detected in the presence of ThzK‐OMe using an anti‐FLAG antibody, confirming its successful incorporation into phages (Figure 4b, left panel). Furthermore, consistent with previous findings showing higher infectivity in phages incorporating UAAs,^[^ ^21^ ^]^ A_6_ThzK‐phage and CA_5_ThzK‐phage exhibited an 81‐fold and 72‐fold increase in infectivity, respectively, compared to phages produced without ThzK‐OMe supplement (Figure 4b, right panel). This also indicated the successful incorporation of ThzK‐OMe into pIII. Next, to install the aldehyde into phages and verify its reactivity, A_6_Thzk‐phages were treated with metal catalysts and biotin probe **18** in a one‐pot reaction. Results showed that only after metal catalyst treatments could the biotinylated pIII band be detected. In contrast, no signals were detected in the absence of catalysts, and for WT phages, regardless of catalyst treatments (Figure 4c). This data confirmed the successful incorporation of aldehydes into the M13 phage particles after Cu(II)/THPTA treatment. Unlike simple recombinant proteins, phages are composed of thousands of proteins assembled in a precise order and are alive. Therefore, a mild and biocompatible chemical approach for post‐functionalization is highly desired in order to maintain their structural integrity and viability. Next, to compare the potential effects of metal catalysts on phage viability and infectivity, phage titers were subsequently measured after catalyst treatments. Notably, the results once again showed that Cu(II)/THPTA had no adverse effects on phage viability compared to PBS treatment. In contrast, prolonged exposure to Pd(II) significantly reduced the infectivity (Figure 4d), indicating the excellent biocompatibility of Cu(II)/THPTA. Overall, through the combined use of the Cu(II)/THPTA and GCE technology, we achieved, for the first time, the site‐specific incorporation of α‐oxo‐aldehydes into the internal positions of peptides displayed on phage particles. This approach successfully expands the diversity of functionalities encoded by phages. Such advancements will facilitate diverse applications, including drug delivery and the selection of novel affinity peptides incorporating unnatural elements.^[^ ^22^ ^]^

### 2.5 Cu(II)/THPTA‐Triggered Thz Cleavage on Living Bacterial Cell Surfaces

**Figure 5:**
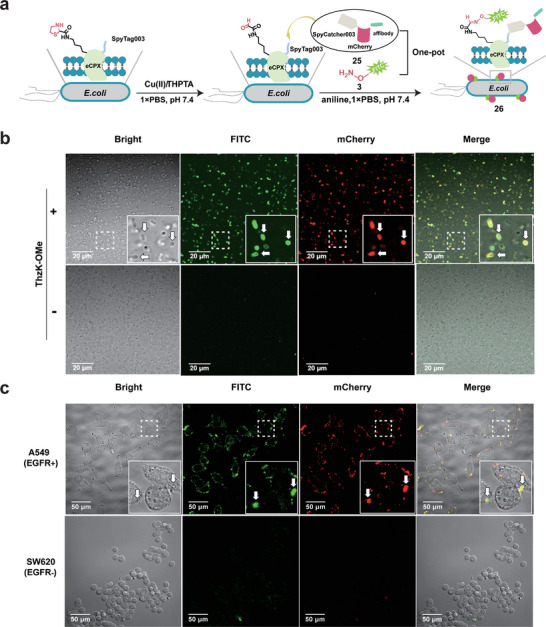
Generation of dual‐color labeled bacterial cells for cancer cell‐selective targeting. a) Schematic illustration of the generation of dual‐labeled bacterial cells via oxime ligation and SpyCatcher/SpyTag Chemistry. The genetically incorporated ThzK in surface‐anchored eCPX in *E. coli* cells can be converted into α‐oxo‐aldehydes by Cu(II)/THPTA, which then react with aminooxy‐FITC probe **3**. Meanwhile, the co‐expressed SpyTag003 can be ligated with a SpyCathcher003‐bearing fusion protein (e.g., **25**) via isopeptide bond formation. The reaction was carried out in the presence of 100 mM aniline in PBS at pH 7.4 in a one‐pot fashion; b) Confocal microscopy images of dual‐labeled *E. coli* cells appended with FITC and mCherry, along with the zoomed‐in views. The one‐pot dual labeling reaction was performed at 37 °C for 4 h. Only in the presence of ThzK‐OMe, green (FITC) and red (mCherry) fluorescent signals were detected, indicating the simultaneous conjugation of FITC and mCherry‐bearing proteins to the bacterial cell surfaces. The white arrows indicate the labeled bacterial cells. Scale bar = 20 µm; c) Confocal microscopy images of A549/SW620 cells treated with dual‐modified *E. coli* cells appended with FITC and affibody_EGFR_‐mCherry‐SpyCatcher003 at 37 °C for 1 h, along with the zoomed‐in views. In contrast to SW620 cells, both green and red fluorescent signals were simultaneously detected and merged in A549 cells, indicating the localization of dual‐color labeled *E. coli* cells and their binding to cancer cell surfaces through the loaded affibody_EGFR_. The white arrows indicate the bound bacterial cells. Scale bar = 50 µm.

Finally, we expanded Cu(II)/THPTA‐triggered Thz cleavage to remodel living bacterial cell surfaces, driven by two key considerations. First, bacterial cells present a more complex living biological system compared to phage particles, making them ideal for showcasing the biocompatibility of our approach. Second, given the extensive applications of bacterial cells in drug delivery and synthetic biology, a biocompatible method capable of simultaneously loading two distinct cargos—such as a drug or fluorescent compound and a targeting moiety—onto cell surfaces would be highly advantageous. Furthermore, to the best of our knowledge, no previous report has demonstrated the site‐specific incorporation of α‐oxo‐aldehydes into bacterial surface proteins. To begin, we initially assessed the cytotoxic effects of selected Thz uncaging metal ions on *E. coli* cells over a 6 h incubation period. Results showed that even at the highest concentration tested (100 µm), Cu(II)/THPTA exhibited minimal toxicity, in stark contrast to Pd(II), which resulted in complete eradication of bacterial cells at the same concentration (Figure S5, Supporting Information). This data highlighted the exceptional biocompatibility of Cu(II)/THPTA with biological systems. Next, to achieve simultaneous dual‐labeling of bacterial cells, we engineered these cells to express a model peptide with an N‐terminal GGGGGX‐FLAG tag sequence for ThzK incorporation at the X position and a SpyTag003 at the C‐terminus on their surfaces using the dual‐display eCPX system.^[^ ^23^ ^]^ Following the scheme shown in **Figure** **5**a, SpyTag003 could only be co‐displayed on the surfaces when ThzK‐OMe was successfully incorporated. Subsequently, an aminooxy‐bearing probe and a SpyCatcher003‐carrying fusion protein were used to generate dual‐labeled bacterial cells via oxime ligation and SpyCatcher/SpyTag chemistry. Successful incorporation of ThzK‐OMe was confirmed via Western blot analysis using an anti‐FLAG antibody (Figure S6, Supporting Information). Next, to expose α‐oxo‐aldehyde groups on the bacterial cell surface, these cells were treated with Cu(II)/THPTA for 2 h, followed by extensive washing with PBS to remove the decaging catalyst. Subsequently, aldehyde‐bearing bacterial cells were co‐incubated with the aminooxy‐FITC **3** and mCherry‐SpyCatcher003 fusion protein **24** (Figure S7, Supporting Information) in PBS for 4 h at 37 °C and then subjected to confocal microscopy analysis. The results showed that only in the presence of ThzK‐OMe could the bacterial cells be simultaneously labeled with FITC and mCherry. In contrast, no fluorescent signals were detected on the bacterial cell surface in the absence of ThzK‐OMe (Figure 5b). This data confirmed the feasibility of the proposed dual labeling scheme shown in Figure 5a. To further highlight the potential utility of our approach, we generated a trifunctional affibody_EGFR_‐mCherry‐SpyCatcher003 fusion protein **25** (Figure S7, Supporting Information). In this fusion protein, the affibody_EGFR_ may endow bacterial cells with the ability to selectively target EGFR‐positive cancer cells once it is attached. To this end, three bacterial samples (**26**, **27**, **28**) were prepared in parallel: sample **26** was conjugated with protein **25**, sample **27** was labeled with protein **24** lacking the targeting moiety, and sample **28** had no labels (See details in Supporting Information). To induce their interactions with cancer cells, all these bacterial cells were co‐incubated with A549 (EGFR^+^) and SW620 (EGFR^−^) cells, respectively, for 1 h followed by confocal microscopy analysis. It was found that only on the surface of A549 cells treated with **26** could green and red fluorescence be simultaneously detected and well merged, which is attributed to the attached dual‐labeled bacterial cells on the surfaces of A549 cells surfaces. In contrast, no fluorescence was observed in SW620 cells treated with any of the three samples, or in A549 cells treated with **27** and **28** (Figure 5c; Figure S8, Supporting Information). Overall, this study indicated that bacterial cells maintained their viability and targeting function well after Cu(II)/THPTA treatment, showcasing the potential utility of the Cu(II)/THPTA‐triggered Thz cleavage in bacterial surface engineering for drug development, targeted therapy, and synthetic biology.^[^ ^24^ ^]^

## 3 Conclusion

In this study, we have not only described Cu(II)/THPTA as a new bioorthogonal catalyst for the Thz bond cleavage reaction under physiological conditions, but also achieved, for the first time, the site‐specific incorporation of α‐oxo‐aldehyde into nanobody 7D12, intact phage particles, and living bacterial cells, expanding the chemical space. Additionally, we also have developed a novel dual labeling scheme for decorating living bacterial surfaces with two distinct functional moieties, which will be useful for the development of bacteria‐based cancer therapy. Traditionally, Thz bond cleavage is used in native chemical ligation (NCL) to release N‐terminal Cys residues for ligation with thioester‐functionalized fragments, typically mediated by copper ions in recently developed methods.^[^ ^25^ ^]^ However, it should be noted that these techniques have been limited to short peptide fragments and conducted under harsh conditions (such as 6 m guanidine hydrochloride) unsuitable for proteins, phage particles, and living cells. To the best of our knowledge, our study represents the first demonstration that copper ions, specifically the Cu(II)/THPTA complex, can be repurposed as a bioorthogonal catalyst to catalyze the Thz cleavage reaction under native conditions and expands it to the scale of proteins and living bacterial cells. Finally, it's also worth noting that Cu(II)/THPTA exhibits much lower cytotoxicity, better water solubility, and a lower price compared to previously used Pd ions. Thus, beyond the showcases presented here, we also believe that it will find broader applications in the future, potentially in living mammalian cells and animals. These applications may include the on‐demand activation of Thz‐caged peptide aldehyde inhibitors for enzyme activity regulation,^[^ ^26^ ^]^ the release of aldehyde drugs from antibody‐drug conjugates for cancer treatment,^[^ ^27^ ^]^ and the manipulation of the chemically synthesized proteins with Thz backbones both in vitro and in vivo,^[^ ^7^, ^28^ ^]^ among others.

## 4 Experimental Section

### Materials and Reagents

Amino acids, coupling reagents, and resins were obtained from Nanjing Peptide Biotechnology Co., Ltd. and GL Biochem (Shanghai) Co., Ltd. All other chemical reagents were of analytical grade and purchased from Bide Pharmatech Co., Ltd, Shanghai Titan Scientific Co., ltd, Xi'an confluore Biological Technology Co., Ltd., and Adamas‐beta. Unless otherwise stated, all solvents and chemicals were used as received without purification. Horseradish Peroxidase (HRP)‐Conjugated Streptavidin was purchased from Genscript Biotech Corporation, and other antibodies were purchased from Daige Biotechnology Co., Ltd. Ni‐NTA agarose was purchased from Tiandi Renhe Biotechnology Co., Ltd. SW620 and A549 cell lines were purchased from Wuhan Shangen Biotechnology Co., Ltd.

### High Performance Liquid Chromatography (HPLC)

Analytical HPLC analyses were performed using a Shimadzu HPLC system equipped with a Jupiter C18 (5 µm, 4.6 × 250 mm) or a Jupiter C4 (5 µm, 4.6 × 250 mm) reverse‐phase column with a flow rate of 1.0 mL min^−1^. Semi‐preparative HPLC was performed using a semi‐preparative HPLC column (Jupiter C18, 5 µm, 10 × 250 mm) on a Shimadzu system with a flow rate of 2 mL min^−1^. Detection was done with a UV–vis‐detector at 220 nm. The buffer system for all the analyses was buffer A H_2_O (containing 0.045% TFA) and buffer B 90% acetonitrile (ACN) in H_2_O (containing 0.045% TFA).

### Mass Spectrometry

Peptide ESI mass spectra data were obtained on a Waters ZQ2000. Protein ESI mass spectra data were obtained on a 6560 Ion mobility QTOF LC/MS mass spectrometer (Agilent Technologies) equipped with an ESI source. The data were acquired in Q‐TOF mode using positive electrospray ionization (ESI+). The raw mass spectrometry data was analyzed using the MassHunter Qual Software package (Agilent Technologies, version B7.0)

### General Plasmid Construction

All plasmids were constructed using the Hieff Clone Plusmulti‐step cloning kit (YEASEN, Shanghai, China) via Gibson assembly. The wild‐type and mutant gene sequences of proteins used in this work were cloned into the pETDuet‐1 vector. The expression of proteins containing the 6 × His tag was controlled by the isopropyl β‐D‐1‐thiogalactopyranoside (IPTG)‐inducible T7 promoter. Newly constructed plasmids were generated by PCR and verified by sequencing (Qingke, Hangzhou, China). The primers used are listed in Table S1 (Supporting Information).

### Western Blot Analysis

Biotin‐labeled proteins were analyzed by 12–18% SDS‐PAGE, and the proteins on the gel were electrotransferred to polyvinylidene fluoride (PVDF) membranes. The membrane was incubated in 15 mL of blocking buffer (5% w/v BSA in 1 × TBST: 100 mm Tris‐HCl, 150 mm NaCl, pH 7.5, 0.1% Tween‐20) at room temperature for 1 h. Subsequently, the membrane was incubated overnight at 4 °C in 15 mL of HRP‐Streptavidin dilution buffer. The membrane was washed three times with 15 mL of TBST, with each wash lasting for 5 min. Biotin‐labeled proteins were visualized using chemiluminescence (ChemiScope6000, Shanghai, China).

### Screening of Metal Ions for Thiazolidine Deprotection and One‐Pot Labeling of Protein

Preparation of stock solutions: A 10 mm stock solution was individually prepared for KCl, CuI, ZnCl_2_, MgCl_2_, Ni(NO_3_)_2_•6H_2_O, MnSO_4_, [Pd(allyl)Cl]_2_, CuSO_4_, CuSO_4_/THPTA and FeCl_3_. Among them, the molar ratio of CuSO_4_/THPTA was 1:3, while the other ions were used alone without ligands. Additionally, except for [Pd(allyl)Cl]_2_, which was initially dissolved in DMSO and then diluted into the reaction buffer, deionized water was used as the solvent for the other metal ions. Meanwhile, a 10 mm stock solution of hydroxylamine reagents, including Biotin‐PEG_3_‐oxyamine **18** and aminooxy‐FITC **3**, was prepared in DMSO, water, or any other suitable solvent.

A typical reaction for the one‐pot labeling of proteins was as follows: To the phosphate buffer, *x* µM of protein, (1–10)*x* µm of metal ion/complex, (2–10)*x* µm of hydroxylamine reagent, and 0.1m were added with the desired final concentration. The solution was then briefly mixed for 5 sec using pipettes. Typically, *x* ranged from 10 to 200. For [Pd(allyl)Cl]_2_ and CuSO_4_/THPTA, the pH was maintained at 7.4, while for the other metal ions, the pH was adjusted to ≈6. The reaction mixture was then incubated overnight in a 37 °C water bath. Aliquoted samples were taken and added to 6× protein loading buffer (375 mm Tris‐HCl, 10% SDS, 30% glycerol, 0.03% bromophenol blue, 600 mm DTT), followed by boiling for 5 min. Subsequently, the samples were subjected to 12–18% SDS‐PAGE analysis. Before staining the gel with Coomassie Brilliant Blue, a gel imager (470 nm) was used to detect in‐gel fluorescence.

### Statistical Analysis

The data were presented as mean ± SD. If not stated otherwise in the figure legends, one out of at least three independent experiments in triplicates was displayed. Statistical analyses and image processing were performed by using ImageJ, Adobe Illustrator 2022, Cytexpert, and GraphPad Prism 8.0.1. The comparison of the two groups was performed by a one‐way ANOVA with Tukey´s multiple comparisons test or Dunnett`s multiple comparisons test. The significant difference was arranged as follows: ^*^ *p*‐value < 0.05, ^**^ *p*‐value < 0.01, ^***^ *p*‐value < 0.001, ^****^ *p* < 0.0001, and ns, not significant.

## Conflict of Interest

The authors declare no conflict of interest.

## Author Contributions

C.M. and G.L. contributed equally to this work. X.B. conceived, designed, supervised the project and wrote the manuscript. C.M. and G.L. performed the experiments, collected, and analyzed the data. J.Y. collected the confocal data of labeled bacterial cells. J.S. and D.Y. offered some help in the cell labeling experiments. D.L. and W.H. completed the synthesis of aminooxy‐FITC and interpreted the NMR data of compounds. S.P. and X.H. assisted with the ESI‐MS analysis of the protein. X.H., B.Y., and X.B. provided assistance with funding and some experimental facilities. All authors discussed the results and commented on the manuscript.

## Supporting information



Supporting Information

## Data Availability

The data that support the findings of this study are available from the corresponding author upon reasonable request.
