# Supplementary material for: Repurposing Copper(II)/THPTA as A Bioorthogonal Catalyst for Thiazolidine Bond Cleavage
Source: Adv Sci (Weinh). 2024 Sep 19;11(42):2408180. doi: 10.1002/advs.202408180 (PMC11558081; doi:10.1002/advs.202408180)
Supplement: Supplementary file 1 — Supporting Information [file ADVS-11-2408180-s001.docx]

Supporting Information

Repurposing Copper/THPTA as A Bioorthogonal Catalyst for Thiazolidine Bond Cleavage

*Chengyun Ma, Guoqing Liu, Juan Yin, Jianan Sun, Disheng Luo, Dechun Yang, Shuo Pang, Wei Hou, Xinya Hemu*, Bang-ce Ye* and Xiaobao Bi**

C. Ma, G. Liu, J. Sun, D. Luo, D. Yang, Prof. W. Hou, Prof. X. Bi
Collaborative Innovation Center of Yangtze River Delta Region Green Pharmaceuticals ,College of Pharmaceutical Sciences
Zhejiang University of Technology
Hangzhou 310014 (China)
*E-mail: xbbi@zjut.edu.cn.

Dr. J. Yin
Zhejiang Yangshengtang Institute of Natural Medication Co., Ltd
Hangzhou 310013(China)

S. Pang, Prof. X. Hemu
School of Traditional Chinese Pharmacy, China Pharmaceutical University
Nanjing 210009 (China)
*E-mail: hemuxinya@cpu.edu.cn.

Prof. BC. Ye
Lab of Biosystem and Microanalysis, State Key Laboratory of Bioreactor Engineering, East China University of Science and Technology
Shanghai 200237 (China)
*E-mail: bcye@ecust.edu.cn

**Table of Contents**

[1. Synthesis of unnatural amino acid ThzK-OMe 3](#_Toc177315582)

[2. Synthesis of peptides 4](#_Toc177315583)

[3. Protein expression and purification 5](#_Toc177315584)

[3.1 Expression and purification of proteins containing ThzK 5](#_Toc177315585)

[3.2 Expression and purification of wild-type proteins without ThzK 6](#_Toc177315586)

[4. Phage expression and purification 7](#_Toc177315587)

[4.1 The Construction and functional validation of the M13KO7ΔpIII helper phage 7](#_Toc177315588)

[4.2 Expression and purification of phage incorporating ThzK 8](#_Toc177315589)

[4.3 Determination of Phage Titer 10](#_Toc177315590)

[4.4 Effect of metal ions on phage activity 10](#_Toc177315591)

[5. Effect of metal ions on the growth of *E. coli* 11](#_Toc177315592)

[6. One-pot dual labeling of living bacterial cells 11](#_Toc177315593)

[7. Cell culture 12](#_Toc177315594)

[8. EGFR-selective cancer cell targeting by 7D12 23 12](#_Toc177315595)

[9. EGFR-selective cancer cell targeting by dual-color labeled living bacterial cells 13](#_Toc177315596)

[10. Supplementary Table 15](#_Toc177315597)

[11. DNA sequence 17](#_Toc177315598)

[12. Supplementary Figures 22](#_Toc177315599)

[13. Unprocessed scans of original blots shown in the main text figures 30](#_Toc177315600)

[14. ESI-MS profile of prepared peptides and ThzK-OMe 33](#_Toc177315601)

[15.NMR spectrum 37](#_Toc177315602)

[References 39](#_Toc177315603)

1. Synthesis of unnatural amino acid ThzK-OMe

**Scheme S1.** The chemical synthesis route for ThzK-OMe.

ThzK-OMe was synthesized according to the previously reported procedure.^1, 2^ Briefly, the key intermediate, *tert*-Butyl 2-(5-(tert-butoxycarbonylamino)-6-methoxy-6-oxohexylcarbamoyl)thiazolidine-3-carboxylate (V), was synthesized as follows: To a stirred solution of *N^a^*-Boc-L-lysine-O-methyl ester Ⅲ (1.3 g, 4.99 mmol, 1.2 eq) in dry CH₂Cl₂ (5 mL) at 0 °C, *N^a^*-Boc-thiazolidine-2-carboxylic acid Ⅳ (0.97 g, 4.16 mmol, 1.0 eq) was added, followed by 1-ethyl-3-(3-dimethylaminopropyl)carbodiimide (0.96 g, 4.99 mmol, 1.2 eq) and 4-dimethylaminopyridine (0.25 g, 2.1 mmol, 0.5 eq). The mixture was stirred and allowed to warm to room temperature overnight, after which the reaction was judged complete by TLC analysis. The reaction mixture was diluted and washed with 1 M HCl, extracted with DCM (2×), and then washed with brine (1×). The organic fractions were dried over Na₂SO₄, filtered, and concentrated under reduced pressure. The residue was purified by flash chromatography (silica, DCM:MeOH (v/v) 20:0 → 19:1) to afford the product as a colorless oil; ^1^H NMR (400 MHz, CDCl_3_) δ 6.94 (s, 1H), 5.40 (s, 1H), 4.94 (s, 1H), 3.91 (s, 1H), 3.68 – 3.48 (m, 2H), 3.42 (s, 3H), 3.09 - 2.83 (m, 3H), 2.82 – 2.63 (m, 1H), 1.58 – 1.35 (m, 2H), 1.33– 1.21 (m, 2H), 1.15 (s, 9H),1.14 (s, 9H), 1.06 – 0.92 (m, 2H). Then, Boc deprotection was performed using 40% trifluoroacetic acid or 2 M HCl in DCM for 1 h. The reaction mixture was concentrated under high vacuum to remove any excess trifluoroacetic acid or HCl. The resulting oil was diluted with H₂O and lyophilized to yield the final product ThzK-OMe Ⅵ as a light yellow gum, which was confirmed by the MS analysis (Expected:275.13; Observed:276.72). Since the Boc-deprotection is quantitative, approximately 10-20 mg of the product was taken and purified using semi-preparative HPLC for NMR analysis. The remaining portion can be used directly in subsequent experiments without further purification. ^1^H NMR (400 MHz, DMSO-*d_6_*) δ 8.51 (t, *J* = 5.6, 1H), 8.62 – 8.34 (m, 2H), 5.14 (s, 1H), 4.02 (t, *J* = 5.6, 1H), 3.75 (s, 3H), 3.57 (dt, *J* = 11.7, 6.0 Hz, 1H), 3.48 – 3.38 (m, 1H), 3.17 – 3.00 (m, 4H), 1.81 – 1.71 (m, 2H), 1.48 – 1.23 (m, 4H).

2. Synthesis of peptides

Peptides were prepared by solid phase peptide synthesis (SPPS) using standard Fmoc chemistry and Rink Amide resin. The synthesis was performed in a 10 mL reaction vessel using the following standard coupling and deprotection methods. Fmoc-protected amino acids (0.5 mM in DMF), HATU (1.475 mM in DMF, 2.95 eq), and DIPEA (1.5 mM in DMF, 3 eq) were used for the coupling reaction, shaken at room temperature for 1-2 h. Deprotection was performed with 20% piperidine in DMF, treating it twice at room temperature for 2 min and 18 min, respectively. Peptides were cleaved from the resin by treatment with a cleavage mixture (95% v/v TFA, 2.5% v/v H_2_O, 2.5% v/v TIPS) for 1-3 h at room temperature. After cleavage, the peptides were precipitated in methyl tert-butyl ether and purified by HPLC. The purified product was then lyophilized to obtain a white powder.

3. Protein expression and purification

3.1 Expression and purification of proteins containing ThzK

The construction of pETDuet-A_6_X-sfGFP plasmid

The previously constructed plasmid pETDuet-sfGFP served as the template. PCR was employed to amplify sfGFP with an N-terminal A_6_X peptide using primers A_6_X-sfGFP F1, A_6_X-sfGFP F2, and A_6_X-sfGFP R, where X represents the site of the amber codon mutation for encoding ThzK. The resulting PCR products were separated by nucleic acid electrophoresis and then recovered from the gel. Subsequently, the recovered product was inserted into the empty pETDuet-1 vector between the NdeI and KpnI sites to generate pETDuet-A_6_X-sfGFP. Colony PCR verification was conducted using two primers, pETDuet YZ F and pETDuet YZ R, and single clones with correct band positions were selected and subjected to sequencing verification. Other site-directed mutagenesis of pETDuet plasmids followed this method.

Expression and purification of A_6_-ThzK-sfGFP **1**

Plasmids pETDuet-A_6_X-sfGFP and pEVOL-MbPylRS-PylT were co-transformed into *E. coli* BL21 (DE3) competent cells, which were then spread on LB-AC plates (containing 100 μg/mL Ampicillin and 50 μg/mL Chloramphenicol) and incubated overnight at 37°C. The following day, a single colony was randomly selected and inoculated into 10 mL of LB-AC medium, then cultured overnight. The overnight culture was subsequently inoculated into 1 L of LB-AC medium and incubated in a 37 °C shaker at 220 rpm. When the OD_600_ reached 0.6-0.8, 2 mM ThzK-OMe, 0.2% L-arabinose, and 1 mM IPTG were added to induce protein expression. After 8 h, cells were harvested by low-temperature centrifugation (4 °C, 8000 rpm, 15 min), then resuspended in lysis buffer A (50 mM NaH_2_PO_4_, 250 mM NaCl, pH 8.0) containing 10 mM imidazole, and sonicated on ice. The cell lysate was centrifuged at 4°C, 10,000 rpm for 40 min and the supernatant was collected. His-tagged sfGFP was purified using Ni-NTA agarose. The agarose was thoroughly washed with lysis buffer containing 10 mM imidazole and 30 mM imidazole to remove non-specifically bound proteins. The protein was then eluted from the agarose using lysis buffer containing 200 mM imidazole. After concentrating the eluate with Millipore ultrafiltration tubes, the buffer was replaced with PBS. The purified protein was analyzed using 12% SDS-PAGE and stored at -80 °C.

Expression and purification of A_5_-ThzK-ubiquitin **2**

The pETDuet-A_5_X-ubiquitin plasmid was constructed from our previously used pETDuet-ubiquitin plasmid using the primers A5X-ubiquitin F1, A5X-ubiquitin F2, A5X-ubiquitin F3, and A5X-ubiquitin R. The expression and purification method of A_5_-ThzK-ubiquitin was the same as for A_6_-ThzK-sfGFP, except that plasmid pETDuet-A_6_X-sfGFP was replaced by plasmid pETDuet-A_5_X-ubiquitin.

Expression and purification of 7D12-Q13ThzK **20**

The pETDuet-7D12-Q13TAG plasmid was constructed from our previously used pETDuet-7D12 plasmid using the primers Q13TAG F1, Q13TAG F2, Q13TAG R1, and Q13TAG R2. The expression and purification method for 7D12-Q13ThzK **20** was the same as for A_6_-ThzK-sfGFP **1**, except that plasmid pETDuet-A_6_X-sfGFP was replaced by plasmid pETDuet-7D12-Q13TAG, and the induction condition for protein expression was changed to 25 °C for 16 h.

3.2 Expression and purification of wild-type proteins without ThzK

Expression and purification of proteins

The plasmid encoding protein of interest (POI) was transformed into *E.coli* BL21 (DE3) competent cells, which were then spread on an LB-A plate (containing 100 μg/mL Ampicillin) and incubated at 37 °C overnight. The following day, a single colony was randomly selected and inoculated into 10 mL of LB-A medium and cultured overnight. The overnight culture was then inoculated into 1 L of LB-A medium and cultured in a 37 °C shaker at 220 rpm. When the OD_600_ reached 0.6-0.8, 1 mM IPTG was added to induce protein expression. The remaining protein purification procedure was almost the same as for A_6_-ThzK-sfGFP. The final purified protein was analyzed by 12% SDS-PAGE and stored at -80 °C.

4. Phage expression and purification

4.1 The Construction and functional validation of the M13KO7ΔpIII helper phage

The M13KO7ΔpIII helper phage plasmid was constructed by deleting the gene encoding the pIII protein in M13KO7. Two primers, ΔpIII F and ΔpIII R, were used for PCR amplification. The reaction products were separated by nucleic acid electrophoresis and then subjected to gel recovery and homologous recombination. Following this, two primers, ΔpIII YZ F and ΔpIII YZ R, were used for colony PCR verification, and single clones with the correct band position were selected for sequencing verification.

To verify that the pIII phenotype in M13KO7ΔpIII has indeed been knocked out, the plasmid M13KO7ΔpIII was transformed into Top10F' competent cells, which were then spread on an LB-K plate (containing 50 μg/mL Kanamycin) and incubated at 37 °C overnight. Subsequently, a single colony was randomly selected and inoculated into LB-K medium, incubated overnight at 30 °C and 220 rpm in a shaker. The bacterial cells were pelleted at 4 °C and 8000 rpm for 30 min, and the supernatant was collected. As a positive control, wild-type M13KO7 phage was also prepared under the same conditions. The supernatant was transferred into a sterile 1.5 mL EP (Eppendorf) tube and heated at 65 °C for 15 min to kill the remaining bacterial cells. Then, 10 µL of the heat-inactivated supernatant was spotted onto top agar containing Top10F'containing 10 µg/mL Tetracycline and placed in a 37 °C incubator overnight. Wild-type M13KO7 phage served as the positive control. It was observed that on the top agar containing Top10F', a cell growth delay area appeared at the position where wild-type M13KO7 phage was spotted, indicating the presence of active phage. However, no cell growth delay area appeared at the spot corresponding to M13KO7ΔpIII, indicating that the functional pIII protein in the M13KO7ΔpIII phage has indeed been completely lost.

To demonstrate the ability of phagemid carrying pIII to complement M13KO7ΔpIII and produce active phage, two phagemids, pSEX81 and pSEX81-FLAG-X-pIII, were separately transformed into Top10F' competent cells containing M13KO7ΔpIII. The pSEX81 phagemid encoded wild-type pIII, while pSEX81-FLAG-X-pIII encoded a pIII protein harboring a FLAG tag and an amber codon mutation at its N-terminal end. A single colony was randomly selected and inoculated into LB-KA medium (containing 50 μg/mL Kanamycin and 100 μg/mL Ampicillin) for overnight culture in a shaker at 37 °C and 220 rpm. Subsequently, 5 mL of the overnight culture was transferred and inoculated into 500 mL of LB-K medium, followed by overnight incubation in a shaker at 30 °C and 220 rpm. The bacterial solution was then centrifuged at 4 °C and 8000 rpm for 30 min. The supernatant was collected and poured into a sterilized beaker. One-fifth volume of 20% PEG8000/2.5 M NaCl solution was then added, and the solution was allowed to settle on ice overnight. Subsequently, it was centrifuged at 10,000 rpm for 30 min at 4 °C to remove the supernatant. The resulting phage pellet was thoroughly dissolved in 5 mL of PBS buffer, then centrifuged at 10,000 rpm for 30 min at 4 °C. The supernatant was transferred into a new sterile centrifuge tube, and one-fifth volume of 20% PEG8000/2.5 M NaCl solution was added again. It was allowed to settle on ice for more than 2 h. Finally, the purified phage particles were harvested by centrifuging at 12,000 rpm for 30 min at 4 °C, and the supernatant was discarded. The gray-white precipitate at the bottom of the centrifuge tube was identified as the phage. An appropriate volume of PBS buffer was added to dissolve it completely, and then the phage expression was analyzed by Western blot. It was found that the pIII protein was present in the phage purified from the *E. coli* culture containing M13KO7ΔpIII and phagemid pSEX81, indicating the presence of fully packaged active phage. However, the pIII protein was not detected in the *E. coli* culture containing M13KO7ΔpIII and phagemid pSEX81-FLAG-X-pIII.

4.2 Expression and purification of phage incorporating ThzK

The Construction of pSEX81-derived phagemids

The plasmid pSEX81 (PROGEN), kept in our laboratory, served as the template. For the construction of the pSEX81-FLAG-A_6_X-pIII phagemid, primers A_6_X- pIII F1, A_6_X-pIII F2, and pIII R were used for PCR amplification of pIII to incorporate the FLAG-A_6_X peptide into its N-terminal end, where 'X' represents the site of the amber stop mutation for encoding ThzK. The resulting PCR products were separated by nucleic acid electrophoresis, followed by gel recovery. The recovered product was then inserted into the empty pSEX81 vector between the NcoI and NheI sites to generate the desired pSEX81-FLAG-A_6_X-pIII. Two primers, pSEX81 YZ F and pSEX81 YZ R, were used for colony PCR verification, and randomly selected single clones with the correct band position were chosen for sequencing verification. Similarly, for the construction of the pSEX81-FLAG-CA_5_X-pIII phagemid, primers CA_5_X F1, CA_5_X F2, CA_5_X F3, and pIII R were used. The process followed was same as to that for the pSEX81-FLAG-A_6_X-pIII construction.

Expression and purification of A_6_X-pIII-phage

The phagemid pSEX81-A_6_X-pIII was transformed into Top10F' competent cells harbouring M13KO7ΔpIII and pEVOL-MbPylRS-PylT-CloDF, which were then spread on an LB-KAC agar plate (containing 50 μg/mL Kanamycin, 100 μg/mL Ampicillin, and 50 μg/mL Chloramphenicol) and incubated overnight at 37 °C. A single colony was randomly selected and inoculated into LB-KAC medium for overnight culture in a shaker at 37 °C and 220 rpm. Then, 5 mL of the overnight culture was inoculated into 500 mL of LB-KAC medium and incubated at 37 °C and 220 rpm until the OD_600_ reached 0.6-0.8. Then, 2 mM ThzK-OMe, 0.2% (w/v) L-arabinose, and 1 mM IPTG were added to induce phage expression at 30 °C and 220 rpm overnight. The bacterial culture was then centrifuged at 4 °C and 8000 rpm for 30 min. The cell pellets were discarded, and the supernatant was transferred into a sterilized beaker. One-fifth volume of 20% PEG8000/2.5 M NaCl solution was added, and the solution was allowed to settle on ice overnight. It was then centrifuged at 10,000 rpm for 30 min at 4 °C to collect the pellet, and the supernatant was discarded. The phage pellet was thoroughly dissolved in 5 mL of PBS buffer. The solution was centrifuged again at 10,000 rpm for 30 min at 4 °C to remove unwanted debris, and the supernatant was transferred into a new sterile centrifuge tube. Subsequently, one-fifth volume of 20% PEG8000/2.5 M NaCl solution was added again and allowed to settle on ice for more than 2 h. The mixture was then centrifuged at 12,000 rpm for 30 min at 4 °C, and the supernatant was removed. The gray-white precipitate at the bottom of the centrifuge tube was identified as the phage. After completely dissolving the phage in an appropriate volume of PBS buffer, an equal volume of 50% glycerol was added, mixed, and frozen at -80°C. Phage expression was analyzed by Western blot.

Expression and purification of CA_5_X-pIII-phage

The expression and purification procedure of pSEX81-CA_5_X-pIII phages was the same as for pSEX81-A_6_X-pIII phages, except that the phagemid was replaced by the phagemids pSEX81-CA_5_X-pIII. Phage expression was analyzed by titer determination.

4.3 Determination of Phage Titer

The 10 μL phage solution was incubated in a 65 °C water bath for 15 min to kill any remaining bacterial cells and then diluted to the appropriate titer with a 10-fold gradient in LB medium. Next, 10 μL of the diluted phage solution was added to 90 μL of TOP10F' cells (OD_600_ = 0.4-0.6) and incubated in a water bath at 37 °C for 1 h. Following the incubation, 10 μL of the mixture was plated onto an LB agar plate and incubated overnight in a 37 °C incubator. The phage titer was determined by counting the colony-forming units (Cfu).

4.4 Effect of metal ions on phage activity

To 100 μL of PBS buffer, 10^10^ phages and 10 μM metal ions were added. For CuSO_4_ alone, the pH was adjusted to 6, while for the other samples, the pH was maintained at 7.4. Phages treated with PBS only served as the control. The solution was thoroughly mixed for 5 sec and then incubated overnight in a 37 °C water bath. One-fifth volume of 20% PEG 8k/2.5 M NaCl solution was added, mixed thoroughly, and placed on ice for more than 2 h. The mixture was then centrifuged at 12,000 rpm for 30 min at 4 °C to remove the supernatant. Subsequently, the phage pellet was dissolved in 100 μL of PBS buffer. This precipitation process was repeated twice to ensure the removal of excess reagent. Finally, the phage was resolubilized in 50 μL of PBS buffer for Western blot analysis and titer determination. Three sets of parallel experiments were conducted to evaluate the effect of metal ions on phage activity. The titer measurement was repeated twice for each group, and the average value was calculated.

$$Phage recovery rate (\%)=\frac{titer of experimental group}{titer of control group}\times100\%$$

5. Effect of metal ions on the growth of *E. coli*

A 2 mL overnight culture of *E. coli* BL21 (DE3) was inoculated into 200 mL of fresh media and incubated at 37 °C with shaking at 220 rpm until reaching an OD_600_ of 0.6-0.7. Subsequently, aliquots of the cultures were taken and transferred into sterile shaking tubes. Then, 10 μM, 20 μM, 50 μM, and 100 μM concentrations of [Pd(allyl)Cl]_2_, CuSO_4_, and CuSO_4_/THPTA were separately added. Cultures without metal ion treatment served as the control. All tubes were placed in a 37 °C incubator with shaking at 220 rpm for 6 h. Samples were collected hourly, and the OD_600_ was measured three times in parallel using a microplate reader. The average value was calculated to obtain the growth curve.

6. One-pot dual labeling of living bacterial cells

Expression and characterization of FLAG-G_5_X-eCPX-Spytag003

Plasmids pETDuet-FLAG-G_5_X-eCPX-Spytag003 and pEVOL-MbPylRS-PylT were co-transformed into *E. coli* BL21(DE3) competent cells, which were then spread on LB-AC plates (containing 50 μg/mL Chloramphenicol and 100 μg/mL Ampicillin), and incubated overnight at 37 °C. A single colony was randomly selected and inoculated into 3 mL of LB-AC medium for overnight. Following this, the overnight culture was inoculated into 50 mL of LB-AC medium at a ratio of 1%, and incubated in a 37 °C shaker at 220 rpm. When the OD_600_ reached 0.8-1.0, 2 mM ThzK, 0.2% L-arabinose, and 1 mM IPTG were added to induce protein expression at 18°C for 18 h. The cells were collected by low-temperature centrifugation, and the precipitated cells were washed twice with PBS buffer and resuspended in 2 mL of PBS buffer (pH 7.4). The expression of FLAG-G_5_X-eCPX-Spytag003 was analyzed by Western blot.

One-pot dual labeling of bacterial cells bearing FLAG-G_5_X-eCPX-Spytag003

To 100 μL of reaction buffer, 1×10^6^ of the aforementioned bacteria in PBS buffer (pH 7.4), 100 μM CuSO_4_/THPTA, 20 μM aminooxy-FITC, 100 μM aniline, and 20 μM SpyCatcher003 fusion protein were added and incubated in a 37 °C water bath for 2 h. Then, the cells were collected by low-temperature centrifugation (4 °C,14,000 rpm, 5 min), and the cell pellets were washed extensively with PBS to remove any unreacted labeling reagents and metal ions. Finally, the cells were resuspended in 100 μL of PBS (pH 7.4) for subsequent analysis by confocal laser scanning microscopy.

7. Cell culture

The A549 (lung cancer) cell line and SW620 (CRC) cell line were cultured in DMEM containing 10% FBS and 1% P/S. All cells were maintained at 37 °C in a cell culture incubator with 5% CO_2_.

8. EGFR-selective cancer cell targeting by 7D12 23

Flow cytometry analysis of labeled cells: A549 cells were seeded at a density of 1×10^5^ cells per 6-well plate (×3). To each well, 1 mL of DMEM culture medium containing 10% FBS and 1% P/S was added, and the plates were then incubated in a 37 °C, 5% CO_2_ incubator. After cell adherence, the cell culture medium was removed, and the cells were washed three times with PBS. Subsequently, the cells were divided into three groups, each separately treated with 100 nM of 7D12 **23**, aminooxy-FITC, and PBS only. All treated cells were then incubated at 37 °C for 30 min, followed by washing with PBS 3-5 times to remove unbound labeling reagents. Finally, flow cytometry was used to analyze the fluorescent labeling of cells.

Cell staining and imaging: A549 and SW620 cells were separately transferred to confocal culture dishes and incubated at 37 °C in a 5% CO_2_ incubator until they adhered. The cell culture medium was then removed, and PBS was added for washing 3 times. Next, 1 mL of 4% paraformaldehyde was added to each culture dish to fix the cells for 15 min. Following fixation, the paraformaldehyde was removed, and PBS was added for washing 3 times. Subsequently, 1 mL of Hoechst 33342 staining solution was added to each culture dish for staining for 15 min. Then, the solution was removed, and the cells were washed 3 times with PBS. Finally, 100 nM of 7D12 **23** was added to each culture dish, and the cells were incubated at 37 °C for 30 min, followed by extensive wash using PBS to completely remove any unreacted labeling reagents. Imaging analysis was performed using confocal laser scanning microscopy.

9. EGFR-selective cancer cell targeting by dual-color labeled living bacterial cells

Bacterial cells samples preparation: Prior to labeling the cancer cells, three bacterial cell samples were prepared following the above procedures. Briefly, to 100 μL of PBS buffer (pH 7.4), 1×10^6^ *E. coli* cells expressing G_5_-ThzK-eCPX-SpyTag003 were added and treated with 100 μM CuSO_4_/THPTA for 2-6 h, followed by extensive wash using PBS to remove any residual CuSO_4_/THPTA. Aliquots of bacterial cells were taken and subjected to one-pot dual labeling reaction. To generate cancer cell-selective *E. coli* cells with dual-color (**26**), bacterial cells were treated with 20 μM aminooxy-FITC, 100 μM aniline, and 20 μM affibody_EGFR_-mCherry-SpyCatcher003 fusion protein **25** in PBS buffer. For the *E. coli* with dual-color but lacking cancer cell selectivity (**27**), they were generated by treating with 20 μM aminooxy-FITC, 100 μM aniline, and 20 μM mCherry-SpyCatcher003 (without affibody) fusion protein **24**. *E. coli* cells without ThzK incorporation **28** served as the control and underwent the same treatment. The dual labeling reaction of bacterial cell surfaces was carried out in a water bath at 37 °C for 4 h. Subsequently, the cells were collected by at 4 °C, 14,000 rpm for 5 min, and the bottom cell pellets were washed extensively using PBS and finally resuspended in 100 μL of PBS buffer (pH 7.4) for subsequent assay.

Cell imaging: A549 and SW620 cells were seeded at a density of 1×10^5^ cells per confocal dish (×3). To each dish, 1 mL of DMEM culture medium containing 10% FBS and 1% P/S was added, and the cells were incubated to adhere at 37 °C in 5% CO_2_. Subsequently, the cell culture medium was removed, and the cells were washed 3 times with PBS for 2 min each time. Next, the above bacterial cells samples (**26**, **27 and 28)** were separately added into cells, and incubated at 37 °C for 1 h. After induction of the interaction between cancer and bacterial cells, the cells were washed 3-5 times with PBS to remove any unbound bacterial cells, followed by confocal microscopy analysis. SW620 cells served as the control and were treated under the same condition as A549 cells.

10. Supplementary Table

**Table S1.** Sequence information for primers

| Primer | Sequence（5’-3’） | | | | |
| --- | --- | --- | --- | --- | --- |
| pETDuet-A_6_X-sfGFP | | | | | |
| A_6_X-sfGFP F1 | | gtataagaaggagatatacatatgatggctgctgcagcg | | | |
| A_6_X-sfGFP F2 | | tgatggctgctgcagcggctgcatagggcattgttagcaaaggtgaaga | | | |
| A_6_X-sfGFP R | | ttctttaccagactcgagggtaccttaatggtgatgatgatggtggctgcctttataca | | | |
| pETDuet-A_5_X-ubiqutin | | | | | |
| A_5_X-ubiquitin F1 | | | | agtataagaaggagatatacatatgatgcatcaccaccat | |
| A_5_X-ubiquitin F2 | | | | agatatacatatgatgcatcaccaccatcaccacgagaacctgtattttcaatgcgct | |
| A_5_X-ubiquitin F3 | | | | agaacctgtattttcaatgcgctgcagctgctgcttagcagatcttcgtcaagacgtt | |
| A_5_X-ubiquitin R | | | | tctttaccagactcgagggtaccttattaaccacctcttagtcttaagacaagat | |
| pETDuet-7D12Q13TAG | | | | | |
| Q13TAG F1 | | gtataagaaggagatatacatatgcaggtcaaattagaagagagtggagggggttcggt | | | |
| Q13TAG F2 | | agagtggagggggttcggtctagacgggcggttcacttcgt | | | |
| Q13TAG R1 | | tggtgatgatggtgatgtgagctaacagtaacctgggtcccttgcccccagtagt | | | |
| Q13TAG R2 | | ttctttaccagactcgagggtaccttaatggtgatgatggtgatgtgagctaacagta | | | |
| pETDuet-affibody-mCherry-SpyCatcher003 | | | | | |
| Affibody F | | | | | taagtataagaaggagatatacatatgctgcaggtagataacaaattcaacaaagaaat |
| Affibody R | | | | | accggaaccaccaccagtttcaggcagt |
| mCherry-SpyC003 F | | | | | actgcctgaaactggtggtggttccggttctggttctggtgtgagcaagggcgaggacg |
| mCherry-SpyC003 R | | | | | ttctttaccagactcgagggtaccttagtggtgatggtggtggtgaatatgagcgt |
| M13KO7ΔpIII | | | | | |
| ΔpIII F | | taaaggctccttttggagcctttttttttggagatttttcatgccagttcttttgggta | | | |
| ΔpIII R | | aaggctccaaaaggagccttta | | | |
| pSEX81-A_6_X-pIII | | | | | |
| A_6_X F1 | | | tgctggcagctcagccggccatggccgattacaaagacgatgatgataaagccgcagc | | |
| A_6_X F2 | | | gatgatgataaagccgcagcggctgccgcgtagaaagatatcagagctgaaactgttga | | |
| A_6_X F3 | | | aaagatatcagagctgaaactgttgaaagttgtttagc | | |

| **Table S1.** Sequence information for primers | | |
| --- | --- | --- |
| Primer | | Sequence（5’-3’） |
| pIII R | | tcgttttattgatgcctcaagctagctgatcattagcacaggcctctagagtcattaag |
| pSEX81-CA_5_X-pIII | | |
| CA_5_X F1 | | tgctggcagctcagccggccatggccgattacaaagacgatgatgataaatgtgcagc |
| CA_5_X F2 | | gatgatgataaatgtgcagctgcggccgcgtagaaagatatcagagctgaaactgttga |
| CA_5_X F3 | | aaagatatcagagctgaaactgttgaaagttgtttagc |
| pIII R | | tcgttttattgatgcctcaagctagctgatcattagcacaggcctctagagtcattaag |
| PCR verification reaction system | | |
| pETDuet YZ F | ttgtacacggccgcataatc | |
| pETDuet YZ R | gctagttattgctcagcgg | |
| pSEX8 YZ F | gcaacgcaattaatgtgagttagctcactc | |
| pSEX8 YZ R | gggcctcttcgctattacgcca | |
| ΔpIII YZ F | taggttggtgccttcgtag | |
| ΔpIII YZ R | agaattaactgaacaccctgaac | |

11. DNA sequence

The DNA sequence of A_6_X-sfGFP

atgatggctgctgcagcggctgcatagggcattgttagcaaaggtgaagaactgtttaccggcgttgtgccgattctggtggaactggatggtgatgtgaatggccataaatttagcgttcgtggcgaaggcgaaggtgatgcgaccaacggtaaactgaccctgaaatttatttgcaccaccggtaaactgccggttccgtggccgaccctggtgaccaccctgacctatggcgttcagtgctttagccgctatccggatcatatgaaacgccatgatttctttaaaagcgcgatgccggaaggctatgtgcaggaacgtaccattagcttcaaagatgatggcacctataaaacccgtgcggaagttaaatttgaaggcgataccctggtgaaccgcattgaactgaaaggtattgattttaaagaagatggcaacattctgggtcataaactggaatataatttcaacagccataatgtgtatattaccgccgataaacagaaaaatggcatcaaagcgaactttaaaatccgtcacaacgtggaagatggtagcgtgcagctggcggatcattatcagcagaataccccgattggtgatggcccggtgctgctgccggataatcattatctgagcacccagagcgttctgagcaaagatccgaatgaaaaacgtgatcatatggtgctgctggaatttgttaccgccgcgggcattacccacggtatggatgaactgtataaaggcagccaccatcatcatcaccattaa

The DNA sequence of A_5_X-ubiquitin

atgatgcatcaccaccatcaccacgagaacctgtattttcaatgcgctgcagctgctgcttagcagatcttcgtcaagacgttaaccggtaaaaccataactctggaagttgaaccatccgataccatcgaaaacgttaaggctaaaattcaagacaaggaaggaattccacctgatcaacaaagattgatctttgccggtaagcagctcgaggacggtagaacgctgtctgattacaacattcagaaggagtcgaccttacatcttgtcttaagactaagaggtggttaa

The DNA sequence of 7D12-Q13TAG

atgcaggtcaaattagaagagagtggagggggttcggtctagacgggcggttcacttcgtctgacatgcgctgcctcgggtcgtacatctcgtagttatgggatgggttggtttcgccaagctccaggtaaggagcgtgagtttgtatctggaatctcatggcgtggtgattctactggctatgctgactccgtgaagggccgttttacaatttcccgcgataatgccaaaaacaccgtagacctgcagatgaattccctgaaacctgaagataccgctatttactattgtgccgccgctgccgggagcgcttggtacggaacgctttatgagtacgactactgggggcaagggacccaggttactgttagctcaggcggtggtggctcccatcaccatcatcaccattggagccaccctcaattcgagaaataa

The DNA sequence of G_5_-sfGFP

atgatgggtggtggcggtggtggcattgttagcaaaggtgaagaactgtttaccggcgttgtgccgattctggtggaactggatggtgatgtgaatggccataaatttagcgttcgtggcgaaggcgaaggtgatgcgaccaacggtaaactgaccctgaaatttatttgcaccaccggtaaactgccggttccgtggccgaccctggtgaccaccctgacctatggcgttcagtgctttagccgctatccggatcatatgaaacgccatgatttctttaaaagcgcgatgccggaaggctatgtgcaggaacgtaccattagcttcaaagatgatggcacctataaaacccgtgcggaagttaaatttgaaggcgataccctggtgaaccgcattgaactgaaaggtattgattttaaagaagatggcaacattctgggtcataaactggaatataatttcaacagccataatgtgtatattaccgccgataaacagaaaaatggcatcaaagcgaactttaaaatccgtcacaacgtggaagatggtagcgtgcagctggcggatcattatcagcagaataccccgattggtgatggcccggtgctgctgccggataatcattatctgagcacccagagcgttctgagcaaagatccgaatgaaaaacgtgatcatatggtgctgctggaatttgttaccgccgcgggcattacccacggtatggatgaactgtataaaggcagccaccatcatcatcaccattaa

The DNA sequence of mCherry-SpyCatcher003

atggtgagcaagggcgaggacgacaacatggccatcatcaaggagttcatgcgcttcaaggtgcacatggagggctccgtgaacggccacgagttcgagatcgagggcgagggcgagggccgcccctacgagggcacccagaccgccaagctgaaggtgaccaagggcggccccctgcccttcgcctgggacatcctgtcccctcagttcatgtacggctccaaggcctacgtgaagcaccccgccgacatccccgactacttgaagctgtccttccccgagggcttcaagtgggagcgcgtgatgaacttcgaggacggcggcgtggtgaccgtgacccaggactcctccctgcaggacggcgagttcatctacaaggtgaagctgcgcggcaccaacttcccctccgacggccccgtaatgcagaagaagaccatgggctgggaggcctcctccgagcggatgtaccccgaggacggcgccctgaagggcgagatcaagcagaggctgaagctgaaggacggcggccactacgacgccgaggtcaagaccacctacaaggccaagaagcccgtgcagctgcccggcgcctacaacgtcaacatcaagctggacatcacctcccacaacgaggactacaccatcgtggaacagtacgagcgcgccgagggccgccactccaccggcggcatggacgagctgtacaagatgtcgtactaccatcaccatcaccatcacgattacgacatcccaacgaccgaaaacctgtattttcagggcgccatggttgataccttatcaggtttatcaagtgagcaaggtcagtccggtgatatgacaattgaagaagatagtgctacccatattaaattctcaaaacgtgatgaggacggcaaagagttagctggtgcaactatggagttgcgtgattcatctggtaaaactattagtacatggatttcagatggacaagtgaaagatttctacctgtatccaggaaaatatacatttgtcgaaaccgcagcaccagacggttatgaggtagcaactgctattacctttacagttaatgagcaaggtcaggttactgtaaatggcaaagcaactaaaggtgacgctcatattcaccaccaccatcaccactaa

The DNA sequence of affibody-mCherry-SpyCatcher003

atgctgcaggtagataacaaattcaacaaagaaatgtgggcggcgtgggaagaaattcgcaacctgccgaacctgaacggctggcagatgaccgcgtttattgcgagcctggtggatgacccaagccaaagcgctaacttgctagcagaagctaaaaagctaaatgatgctcaggcgccgaaagtagacggaggtggaggatctggtggactgcctgaaactggtggtggttccggttctggttctggtgtgagcaagggcgaggacgacaacatggccatcatcaaggagttcatgcgcttcaaggtgcacatggagggctccgtgaacggccacgagttcgagatcgagggcgagggcgagggccgcccctacgagggcacccagaccgccaagctgaaggtgaccaagggcggccccctgcccttcgcctgggacatcctgtcccctcagttcatgtacggctccaaggcctacgtgaagcaccccgccgacatccccgactacttgaagctgtccttccccgagggcttcaagtgggagcgcgtgatgaacttcgaggacggcggcgtggtgaccgtgacccaggactcctccctgcaggacggcgagttcatctacaaggtgaagctgcgcggcaccaacttcccctccgacggccccgtaatgcagaagaagaccatgggctgggaggcctcctccgagcggatgtaccccgaggacggcgccctgaagggcgagatcaagcagaggctgaagctgaaggacggcggccactacgacgccgaggtcaagaccacctacaaggccaagaagcccgtgcagctgcccggcgcctacaacgtcaacatcaagctggacatcacctcccacaacgaggactacaccatcgtggaacagtacgagcgcgccgagggccgccactccaccggcggcatggacgagctgtacaagatgtcgtactaccatcaccatcaccatcacgattacgacatcccaacgaccgaaaacctgtattttcagggcgccatggttgataccttatcaggtttatcaagtgagcaaggtcagtccggtgatatgacaattgaagaagatagtgctacccatattaaattctcaaaacgtgatgaggacggcaaagagttagctggtgcaactatggagttgcgtgattcatctggtaaaactattagtacatggatttcagatggacaagtgaaagatttctacctgtatccaggaaaatatacatttgtcgaaaccgcagcaccagacggttatgaggtagcaactgctattacctttacagttaatgagcaaggtcaggttactgtaaatggcaaagcaactaaaggtgacgctcatattcaccaccaccatcaccactaa

The DNA sequence of A_6_X-pIII

gattacaaagacgatgatgataaagccgcagcggctgccgcgtagaaagatatcagagctgaaactgttgaaagttgtttagcaaaatcccatacagaaaattcatttactaacgtctggaaagacgacaaaactttagatcgttacgctaactatgagggctgtctgtggaatgctacaggcgttgtagtttgtactggtgacgaaactcagtgttacggtacatgggttcctattgggcttgctatccctgaaaatgagggtggtggctctgagggtggcggttctgagggtggcggttctgagggtggcggtactaaacctcctgagtacggtgatacacctattccgggctatacttatatcaaccctctcgacggcacttatccgcctggtactgagcaaaaccccgctaatcctaatccttctcttgaggagtctcagcctcttaatactttcatgtttcagaataataggttccgaaataggcagggggcattaactgtttatacgggcactgttactcaaggcactgaccccgttaaaacttattaccagtacactcctgtatcatcaaaagccatgtatgacgcttactggaacggtaaattcagagactgcgctttccattctggctttaatgaggatttatttgtttgtgaatatcaaggccaatcgtctgacctgcctcaacctcctgtcaatgctggcggcggctctggtggtggttctggtggcggctctgagggtggtggctctgagggtggcggttctgagggtggcggctctgagggaggcggttccggtggtggctctggttccggtgattttgattatgaaaagatggcaaacgctaataagggggctatgaccgaaaatgccgatgaaaacgcgctacagtctgacgctaaaggcaaacttgattctgtcgctactgattacggtgctgctatcgatggtttcattggtgacgtttccggccttgctaatggtaatggtgctactggtgattttgctggctctaattcccaaatggctcaagtcggtgacggtgataattcacctttaatgaataatttccgtcaatatttaccttccctccctcaatcggttgaatgtcgcccttttgtctttggcgctggtaaaccatatgaattttctattgattgtgacaaaataaacttattccgtggtgtctttgcgtttcttttatatgttgccacctttatgtatgtattttctacgtttgctaacatactgcgtaataaggagtcttaa

The DNA sequence of FLAG-CA_5_X-pIII

gattacaaagacgatgatgataaatgtgcagctgcggccgcgtagaaagatatcagagctgaaactgttgaaagttgtttagcaaaatcccatacagaaaattcatttactaacgtctggaaagacgacaaaactttagatcgttacgctaactatgagggctgtctgtggaatgctacaggcgttgtagtttgtactggtgacgaaactcagtgttacggtacatgggttcctattgggcttgctatccctgaaaatgagggtggtggctctgagggtggcggttctgagggtggcggttctgagggtggcggtactaaacctcctgagtacggtgatacacctattccgggctatacttatatcaaccctctcgacggcacttatccgcctggtactgagcaaaaccccgctaatcctaatccttctcttgaggagtctcagcctcttaatactttcatgtttcagaataataggttccgaaataggcagggggcattaactgtttatacgggcactgttactcaaggcactgaccccgttaaaacttattaccagtacactcctgtatcatcaaaagccatgtatgacgcttactggaacggtaaattcagagactgcgctttccattctggctttaatgaggatttatttgtttgtgaatatcaaggccaatcgtctgacctgcctcaacctcctgtcaatgctggcggcggctctggtggtggttctggtggcggctctgagggtggtggctctgagggtggcggttctgagggtggcggctctgagggaggcggttccggtggtggctctggttccggtgattttgattatgaaaagatggcaaacgctaataagggggctatgaccgaaaatgccgatgaaaacgcgctacagtctgacgctaaaggcaaacttgattctgtcgctactgattacggtgctgctatcgatggtttcattggtgacgtttccggccttgctaatggtaatggtgctactggtgattttgctggctctaattcccaaatggctcaagtcggtgacggtgataattcacctttaatgaataatttccgtcaatatttaccttccctccctcaatcggttgaatgtcgcccttttgtctttggcgctggtaaaccatatgaattttctattgattgtgacaaaataaacttattccgtggtgtctttgcgtttcttttatatgttgccacctttatgtatgtattttctacgtttgctaacatactgcgtaataaggagtcttaa

The DNA sequence of FLAG-G_5_X-eCPX-Spytag003

atgaagaagatcgcatgtctgagcgcactggccgccgttttagcatttaccgcaggtaccagcgtggccggtcagagcggtcaggactataaagatgatgatgataagggcggtggtggtggctagggtggtggttcaggtggtggtagtggtggtggttctggcggtggtagtggcggtggttcaggaggtcagagcggtcaaagcggtgattataataaaaaccagtactacggcatcaccgccggtccggcatatcgtattaatgattgggccagcatttacggcgtggttggtgtgggctatggcaaatttcagaccaccgaatatccgacctataaacatgataccagcgattatggtttcagctatggtgcaggtctgcagtttaatccgatggaaaatgttgcactggattttagctacgaacagagccgcattcgcagtgttgatgttggcacctggattctgagtgtgggttatcgttttggtagtaaaagccgtcgtgccaccagcaccgttaccggtggttatgcacagagcgatgcacagggccagatgaataaaatgggcggctttaatctgaagtaccgttatgaagaagacaacagcccgctgggtgtgattggtagctttacctataccgaaaaaagccgtaccgcaagtggtggccagagtggtcagcgtggtgttcctcatattgttatggttgatgcatacaagcgttacaaataa

12. Supplementary Figures


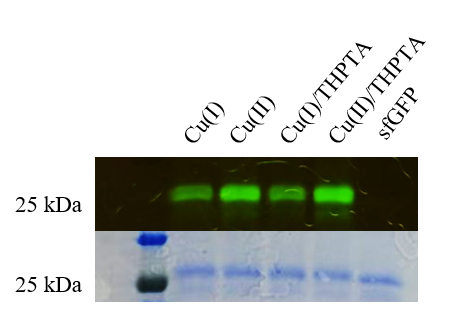


**Figure S1**. Comparison of the efficiency of Cu(I) and Cu(II) in Thz bond cleavage. To a solution of sfGFP-ThzK **1** (1 eq., 50 µM) in PBS buffer (pH 7.4), catalysts (2 eq., 100 µM), FITC probe **3** (2 eq., 100 µM), aniline (2 eq., 100 µM) were added subsequently. The solution was mixed and kept at 37 °C for overnight. Finally, the protein was separated on an SDS-PAGE gel and analyzed by fluorescent signal detection (upper) and Coomassie Blue staining (lower). Based on the fluorescent intensity, it was found that Cu(II) and Cu(II)/THPTA exhibited higher efficiency than Cu(I) and Cu(I)/THPTA in catalyzing the reaction.


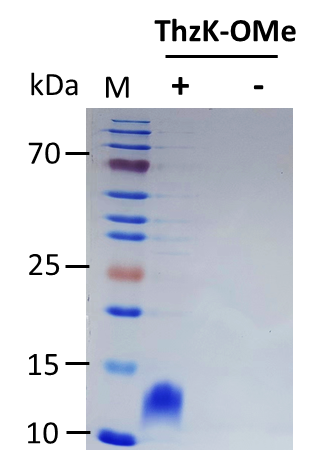


**Figure S2.** SDS-PAGE analysis of purified 7D12-Q13TAG in the presence and absence of ThzK-OMe.


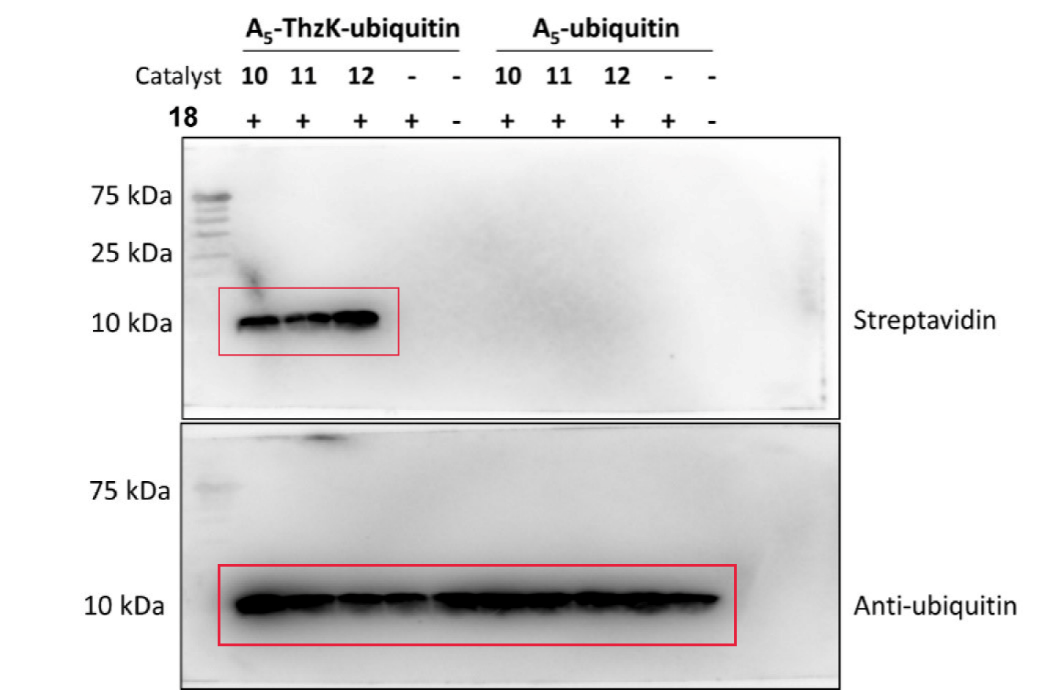


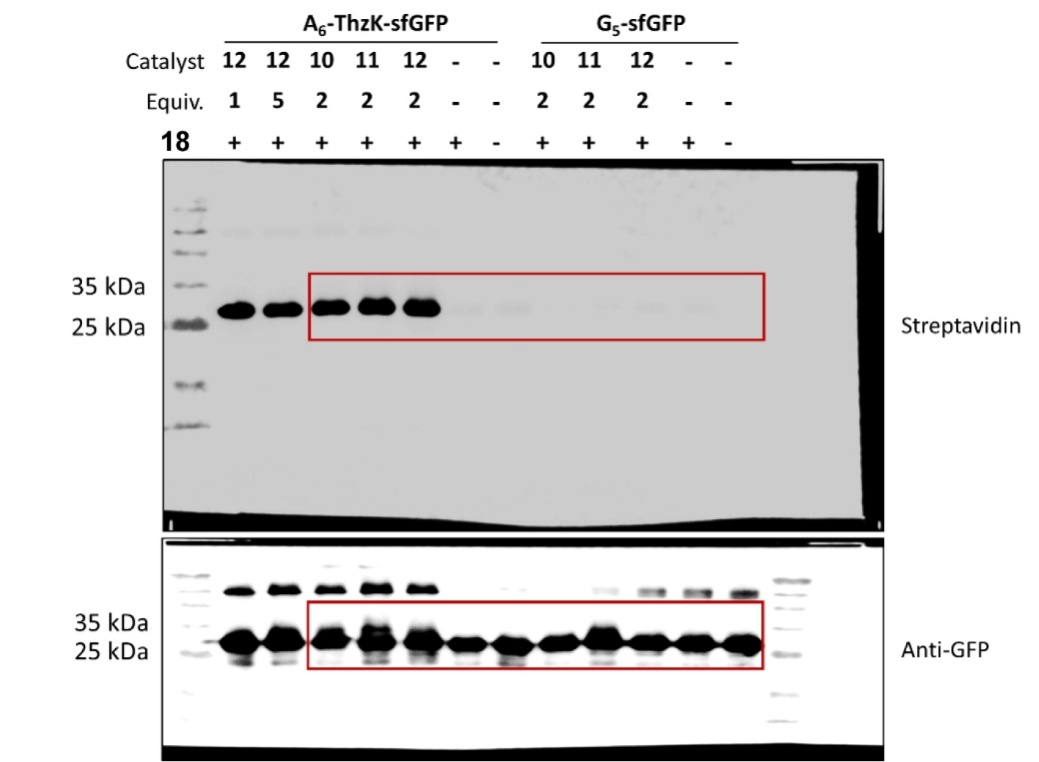


**Figure S3.** Unprocessed scans of the original blots for the detection of biotin-labeled ubiquitin (upper) and sfGFP (lower) are shown. Only ubiquitin and sfGFP incorporating ThzK were successfully labeled using Streptavidin-HRP conjugate. The protein bands detected with anti-ubiquitin and anti-GFP antibodies served as controls to indicate the loaded samples. This shows that biotinylated protein could only be detected in the presence of both ThzK and metal catalysts—Pd(II) **10**, Cu(II) **11**, and Cu(II)/THPTA **12**. The desired band regions are highlighted by the red rectangle.


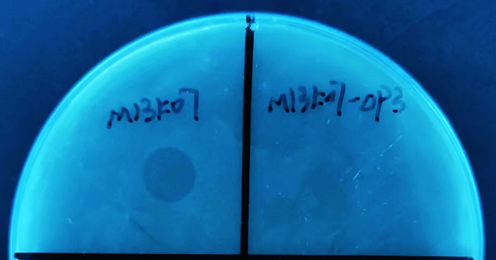


**Figure S4.** Image of Top10F' cells infected with wild-type M13KO7 (left) and M13KO7ΔpIII (right), respectively, illustrating that on top agar containing Top10F', areas of cell growth delay appeared at the locations of wild-type M13KO7 phage (left), indicating the presence of active phage. However, no cell growth delay areas were observed at the spots corresponding to M13KO7ΔpIII (right), suggesting that the functional pIII protein in the M13KO7ΔpIII phage has indeed been completely lost.


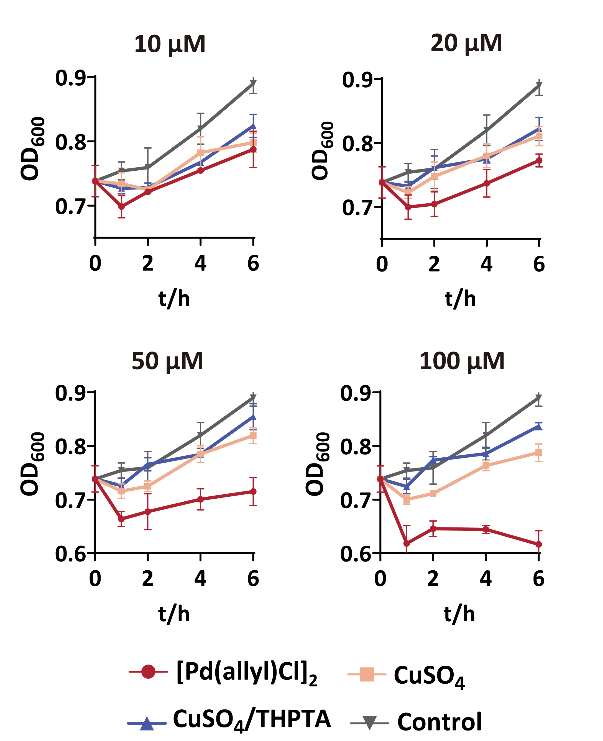


**Figure S5.** Effects of different metal ions on the growth of *E. coli* at four different concentrations during a 6 h period. At the highest concentration tested (100 μM), Cu(II)/THPTA exhibited minimal toxicity, in stark contrast to Pd(II), which resulted in complete eradication of bacterial cells at the same concentration.


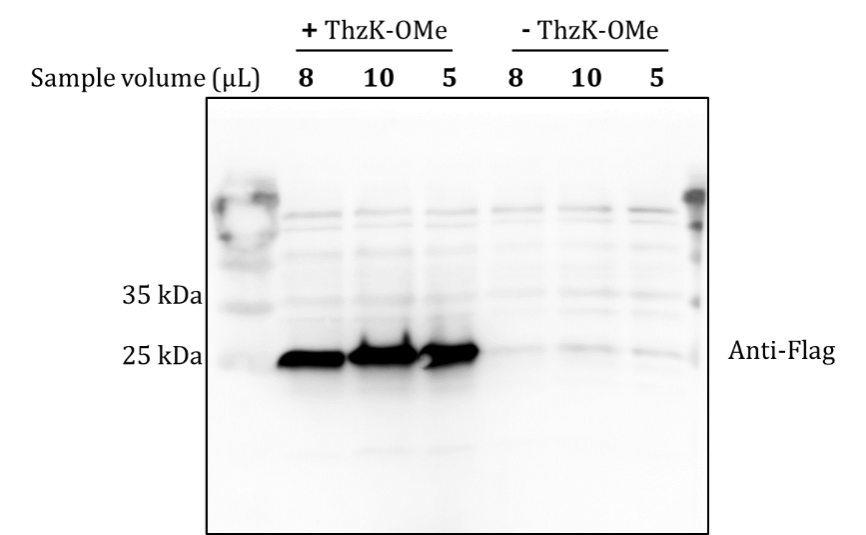


**Figure S6.** Western blot analysis of the expression of FLAG-G_5_X-eCPX-Spytag003 in the presence and absence of ThzK-OMe. It was shown that only in the presence of ThzK-OMe, the full-length protein could be detected by using anti-FLAG antibody.


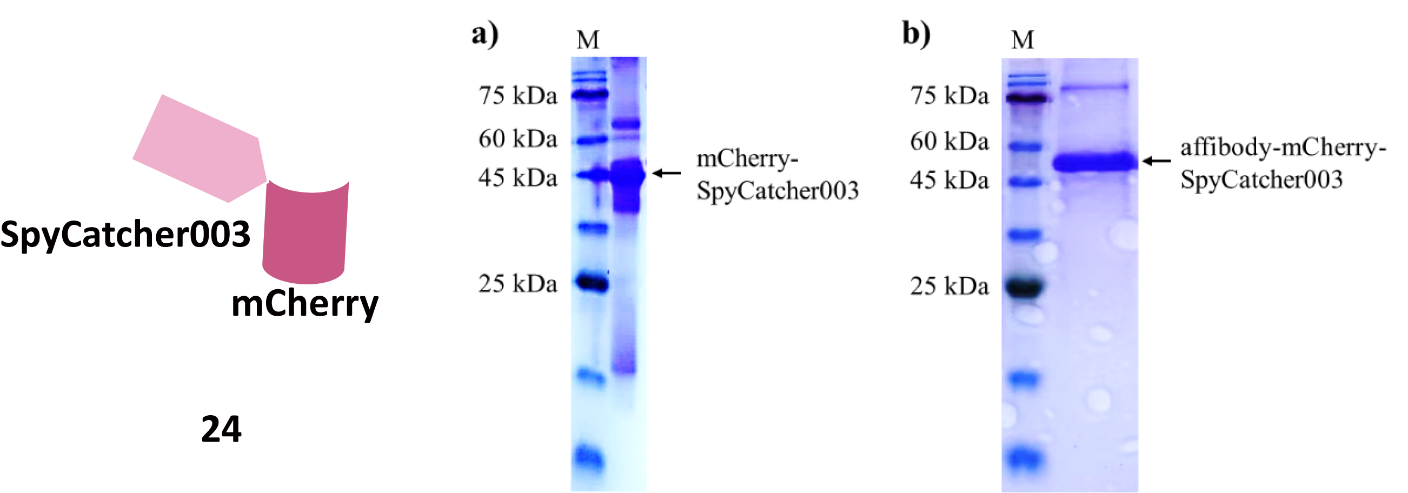


**Figure S7.** SDS-PAGE analysis of purified SpyCatcher003 fusion proteins **24** (a) and **25** (b).


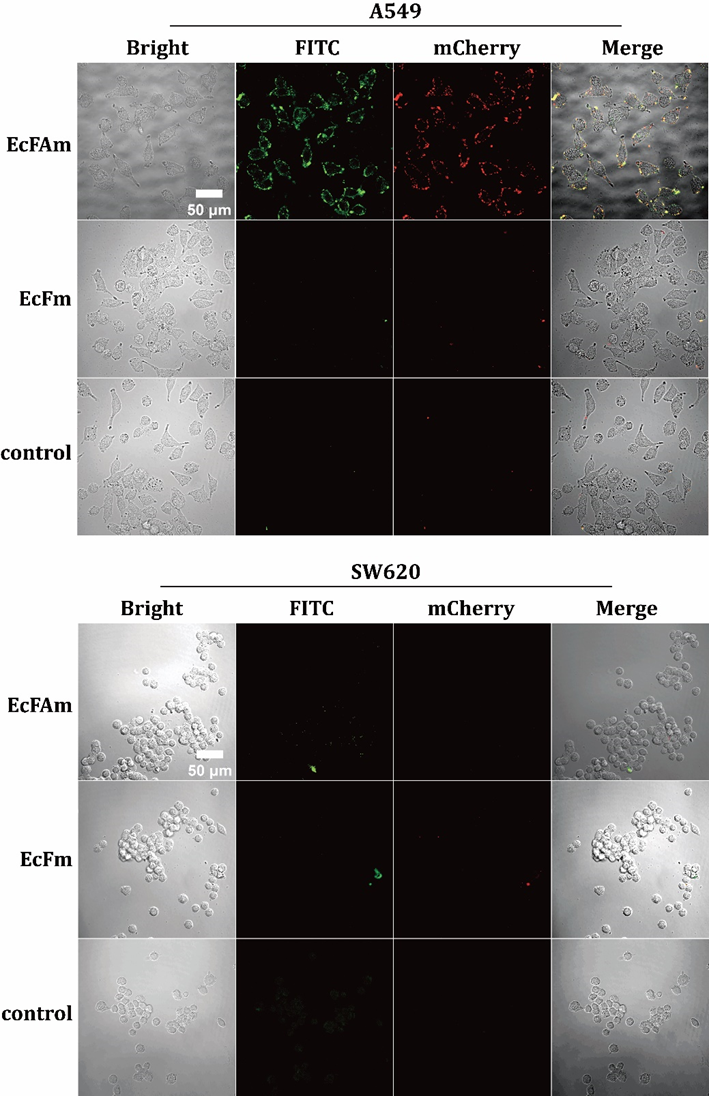

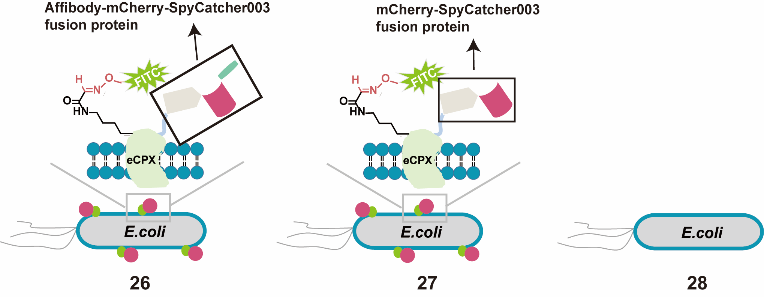

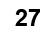

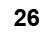

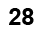

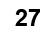

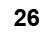

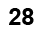


**Figure S8.** Representative confocal images of A549/SW620 cells after incubation with dual-color labeled *E. coli* cells. Only on the A549 cell membrane surface treated with **26** could green and red fluorescent signals be detected. No obvious signals were observed on A549 cells treated by **27** and **28**, and SW620 cells treated by **26**, **27** and **28**. Furthermore, the green and red fluorescence signals were perfectly co-localized, demonstrating that dual-color labeled **26** retains EGFR-targeting ability. Scale bars represent 50 µm.

13. Unprocessed scans of original blots shown in the main text figures


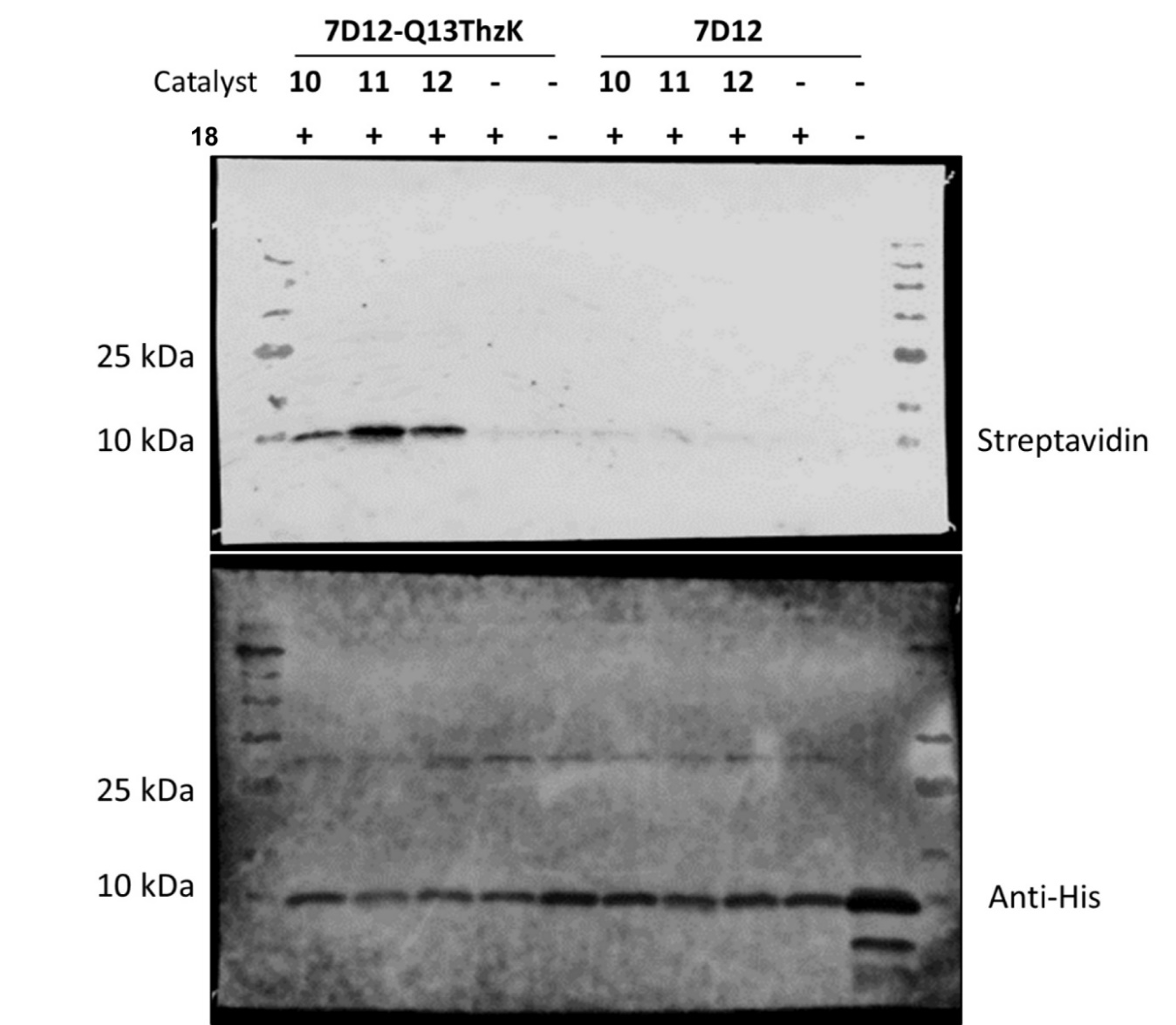


**Figure S9.** Unprocessed scans of original blots for Figure 3b. Western blot analysis of chemically modified 7D12 with biotin. Compared to the wild type 7D12 group, biotin-labeled 7D12 **22** could only be detected in 7D12-Q13ThzK treated by Pd(II) **10**, Cu(II) **11** and Cu(II)/THPTA **12** (upper panel); WB: Streptavidin-HRP conjugate and Anti-His tag antibody were used.


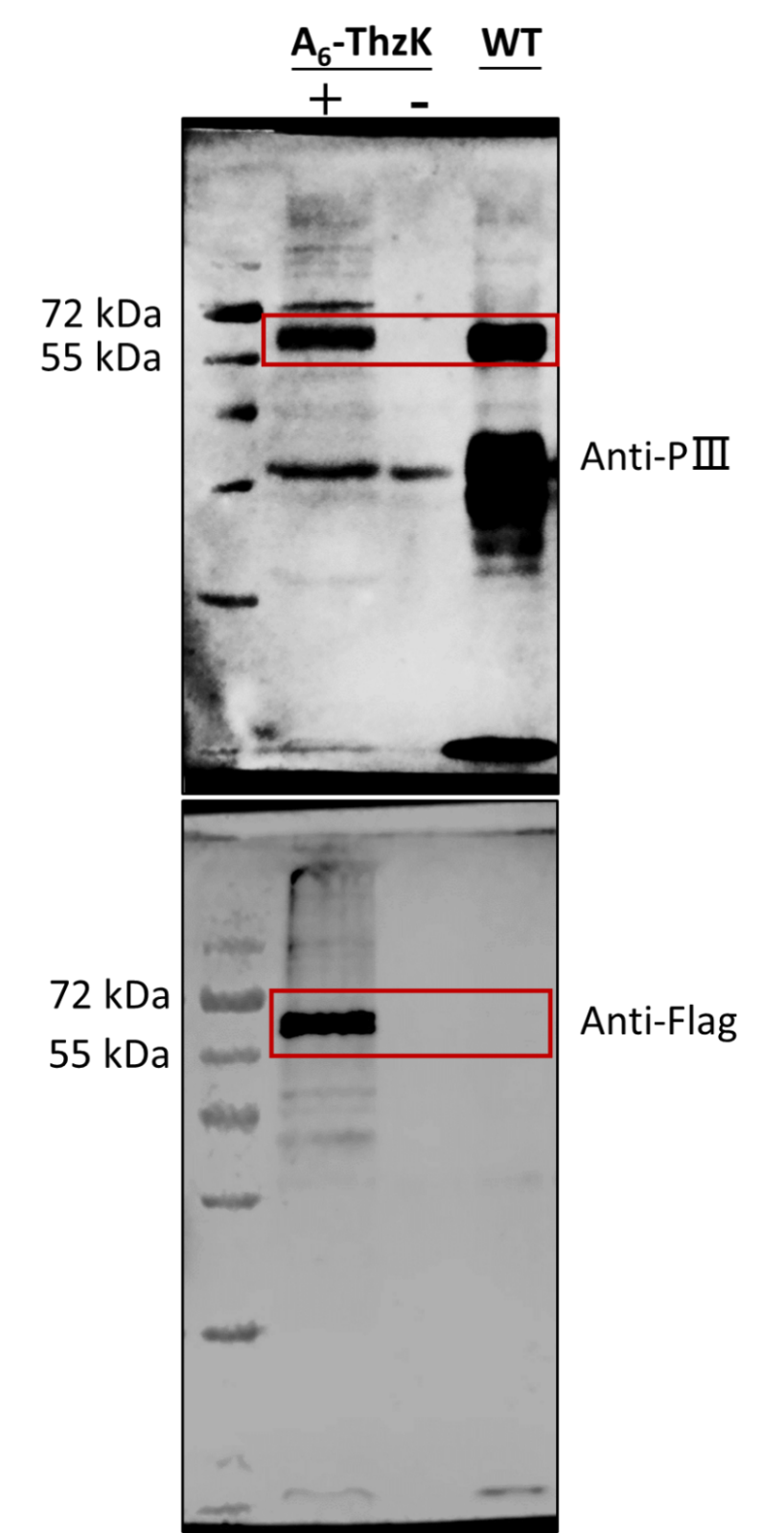


**Figure S10.** Unprocessed scans of original blots for Figure 4b. Western blot detection of ThzK-OMe incorporation into phages. Only in the presence of ThzK-OMe were both pIII protein and FLAG tag simultaneously detected in the A_6_-ThzK phage using anti-pIII and anti-FLAG antibodies, indicating the expression of full-length FLAG-A_6_ThzK-pIII. No FLAG signal was detected in the WT phages (lower panel). The desired band region is highlighted by the red rectangle.


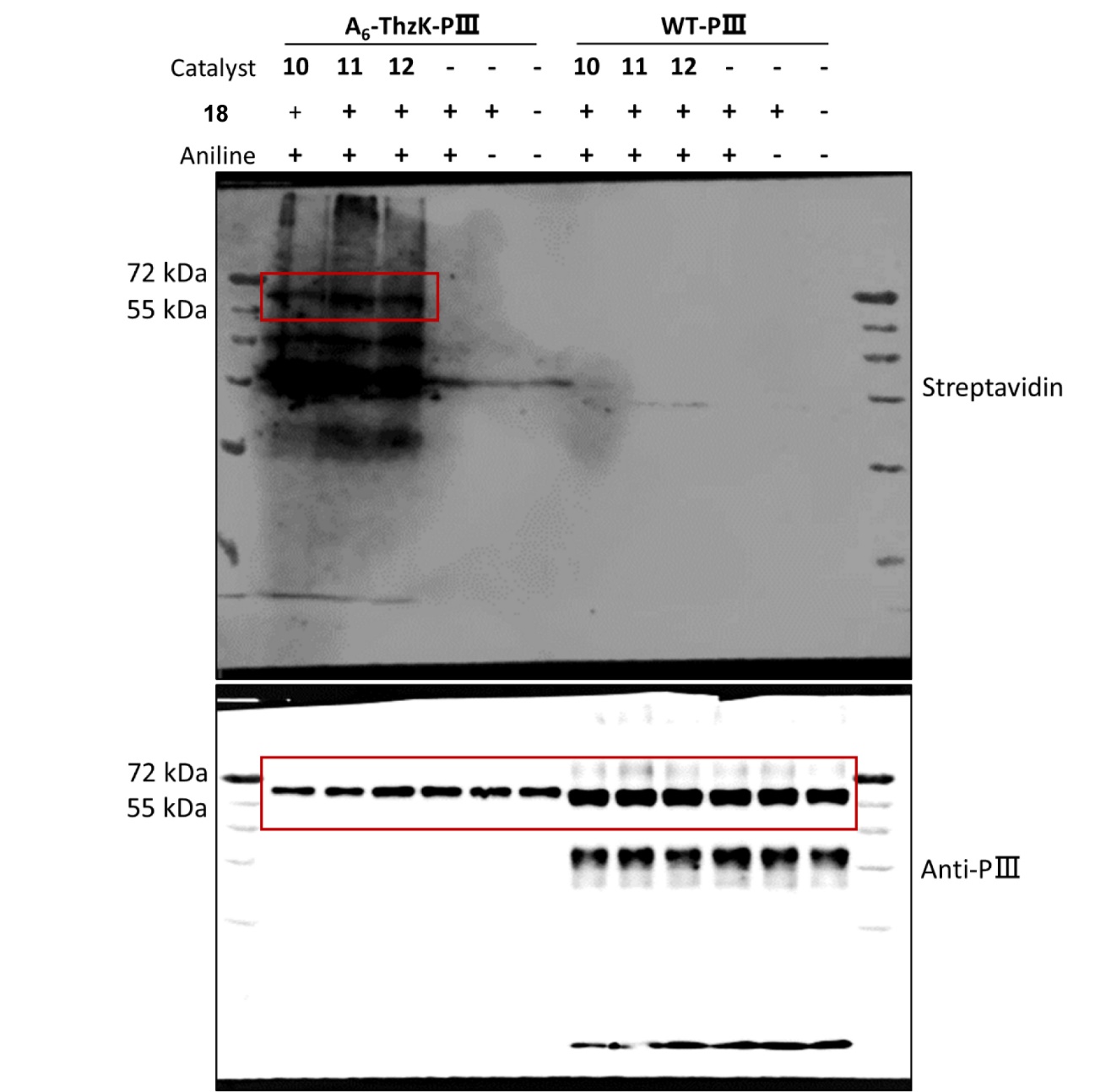


**Figure S11.** Unprocessed scans of original blots for Figure 4c. Western blot analysis of phages labeled with biotin probe **18**. In contrast to WT phages, biotinylated pIII bands were only detected in A_6_ThzK-pIII phages treated with Pd(II) **10**, Cu(II) **11**, or Cu(II)/THPTA **12** by using Streptavidin-HRP conjugate and anti-pIII antibody.

14. ESI-MS profile of prepared peptides and ThzK-OMe


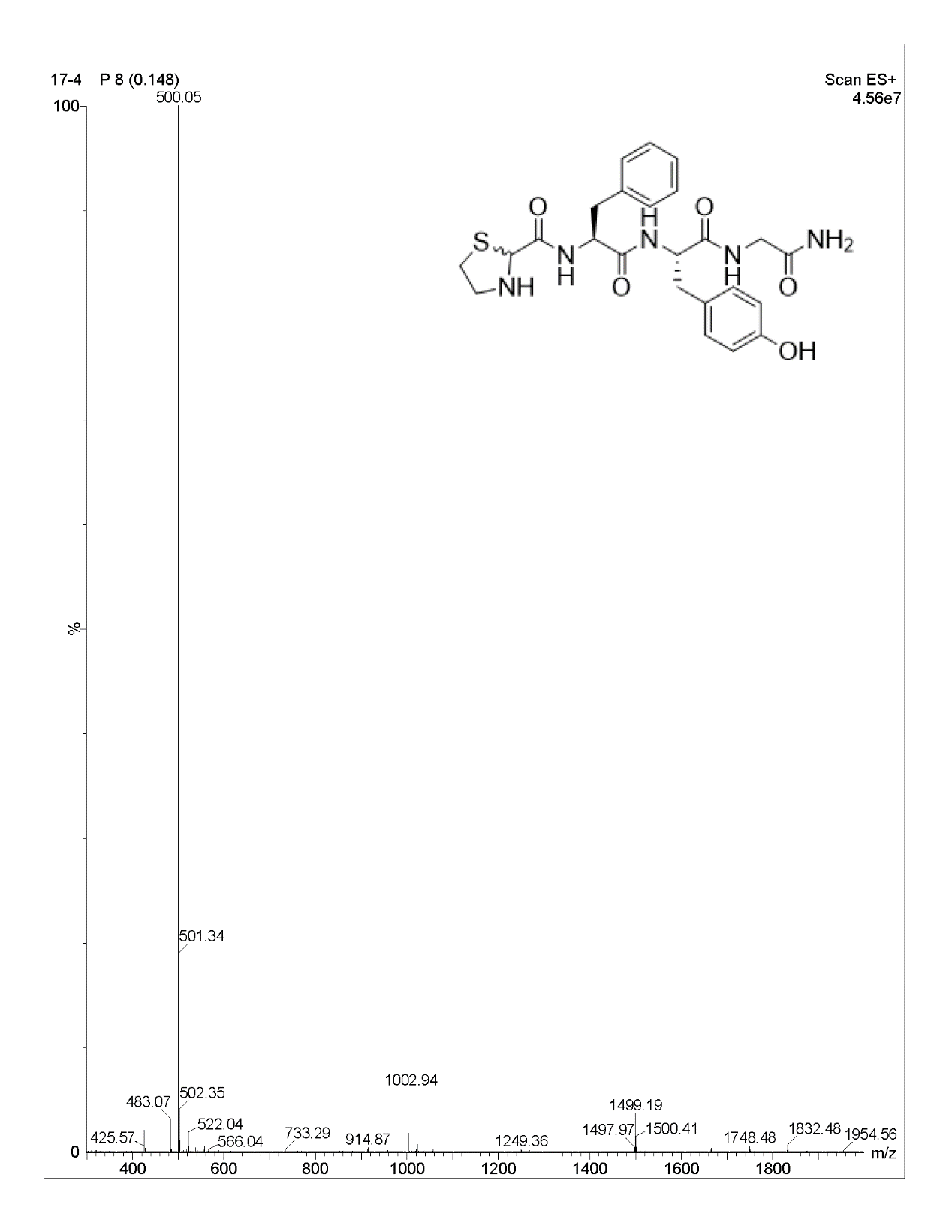


**Figure S12**. ESI-MS analysis of the starting material peptide Thz-FYG-NH_2_. The calculated mass is 499.19, while the observed mass is 500.05.


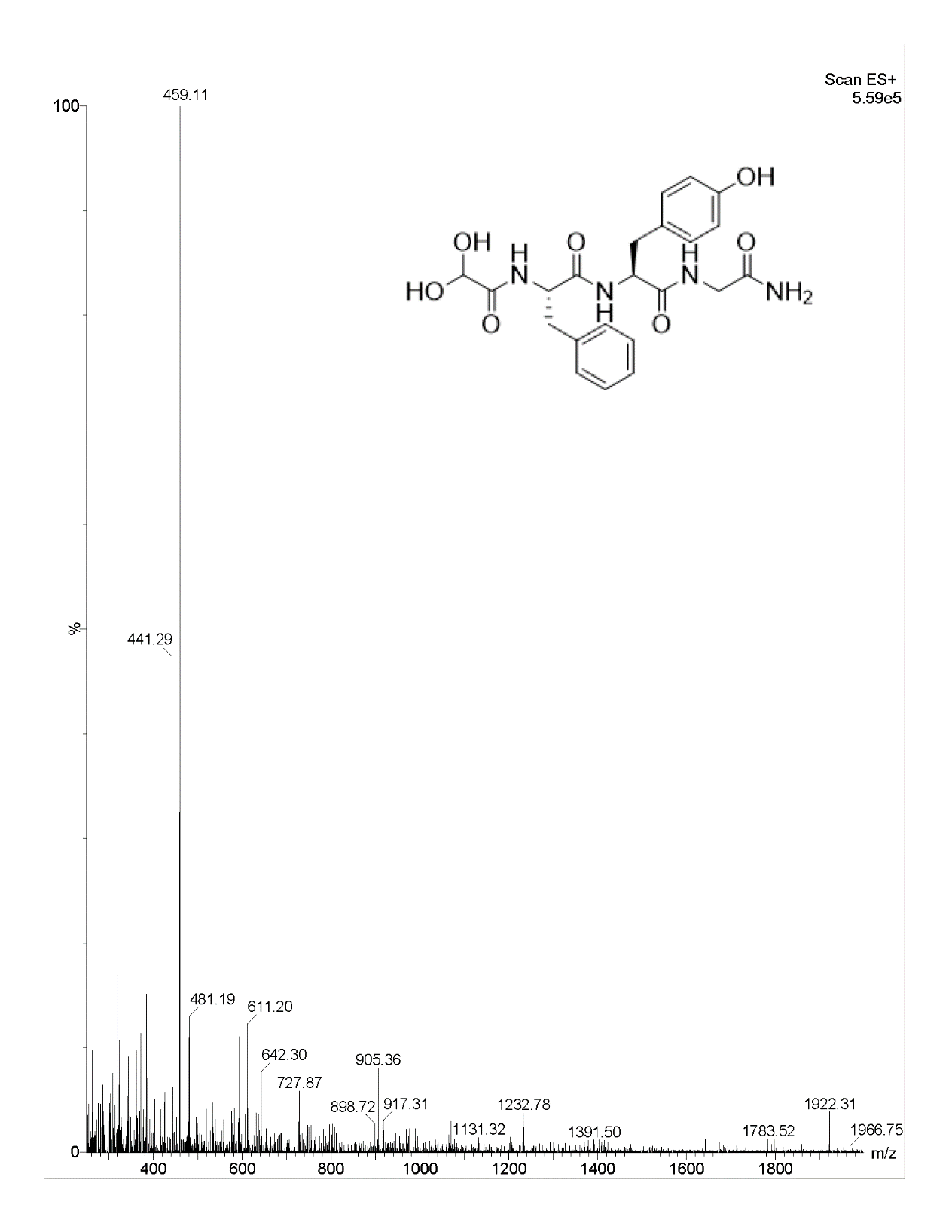


**Figure S13.** ESI-MS analysis of CHO-FYG-NH_2_. The observed masses, 441.29 and 459.11, are matched with the expected masses of 440.17 and 458.18 (the hydrated form).


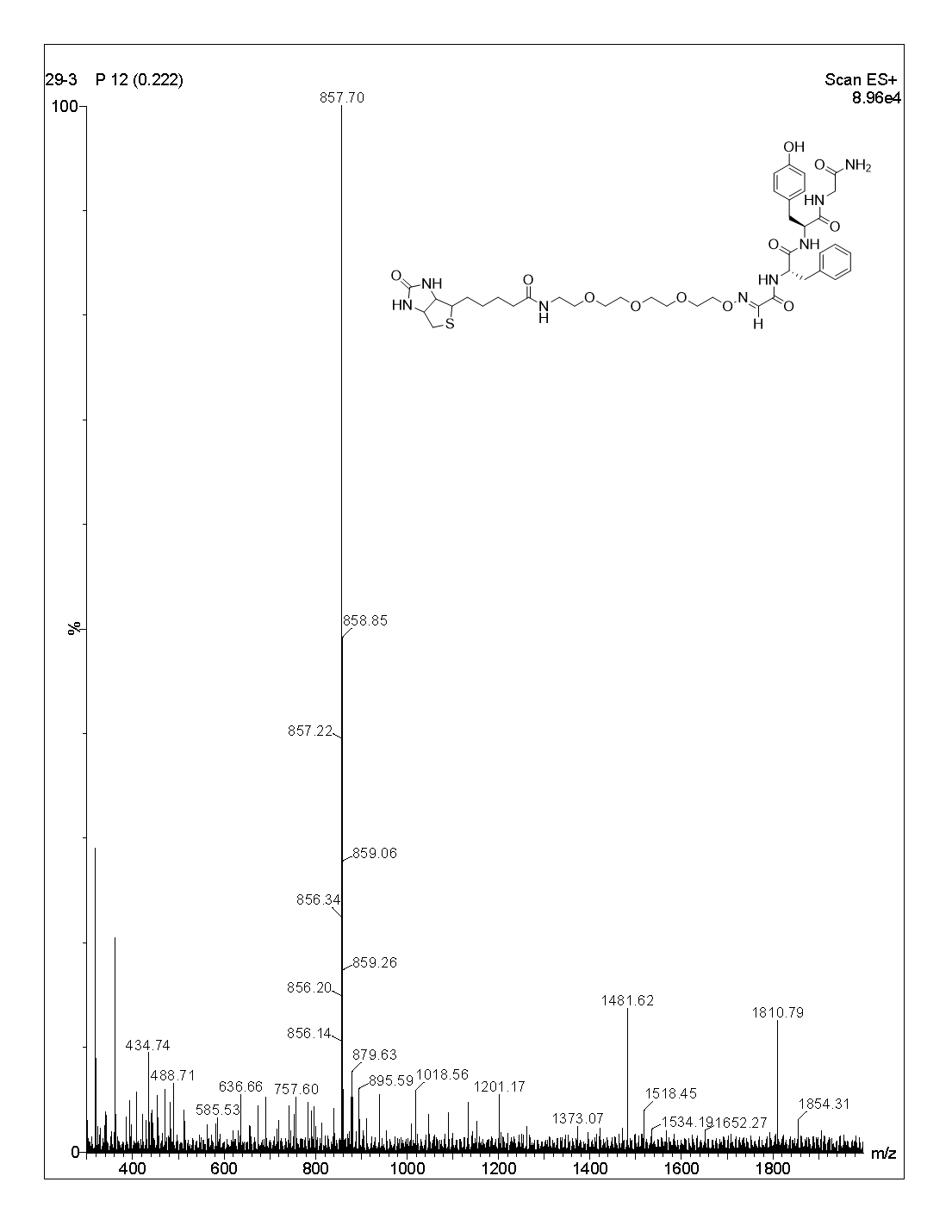


**Figure S14.** ESI-MS analysis of the biotinylated peptide Biotin-FYG-NH_2_. The observed mass is 857.70, while the calculated mass is 856.38.

**Figure S15.** ESI-MS analysis of ThzK-OMe. The observed mass is 276.72, while the calculated mass is 275.13.

# 15.NMR spectrum

**
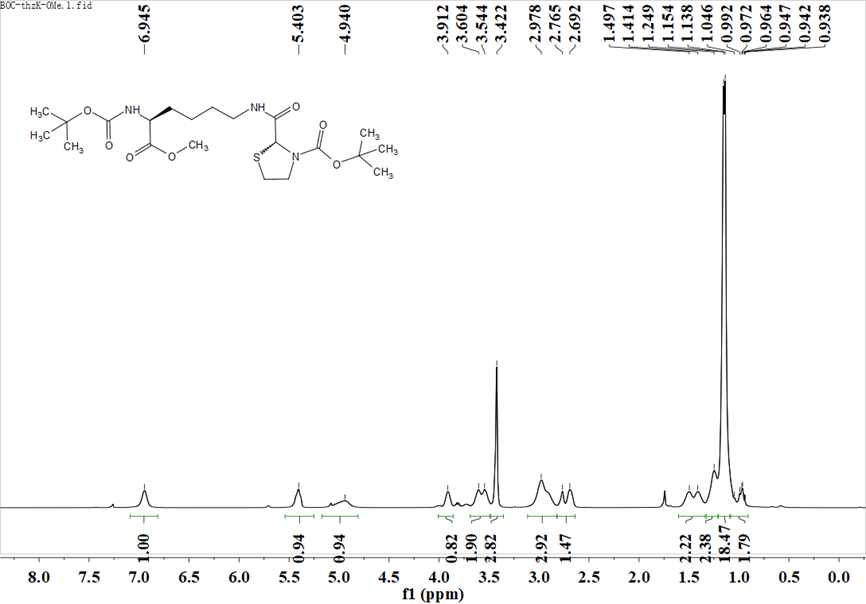
**

**Figure S16.** ^1^H NMR spectrum of *tert*-Butyl 2-(5-(tert-butoxycarbonylamino)-6-methoxy-6-oxohexylcarbamoyl)thiazolidine-3-carboxylate (400 MHz, CDCl_3_).


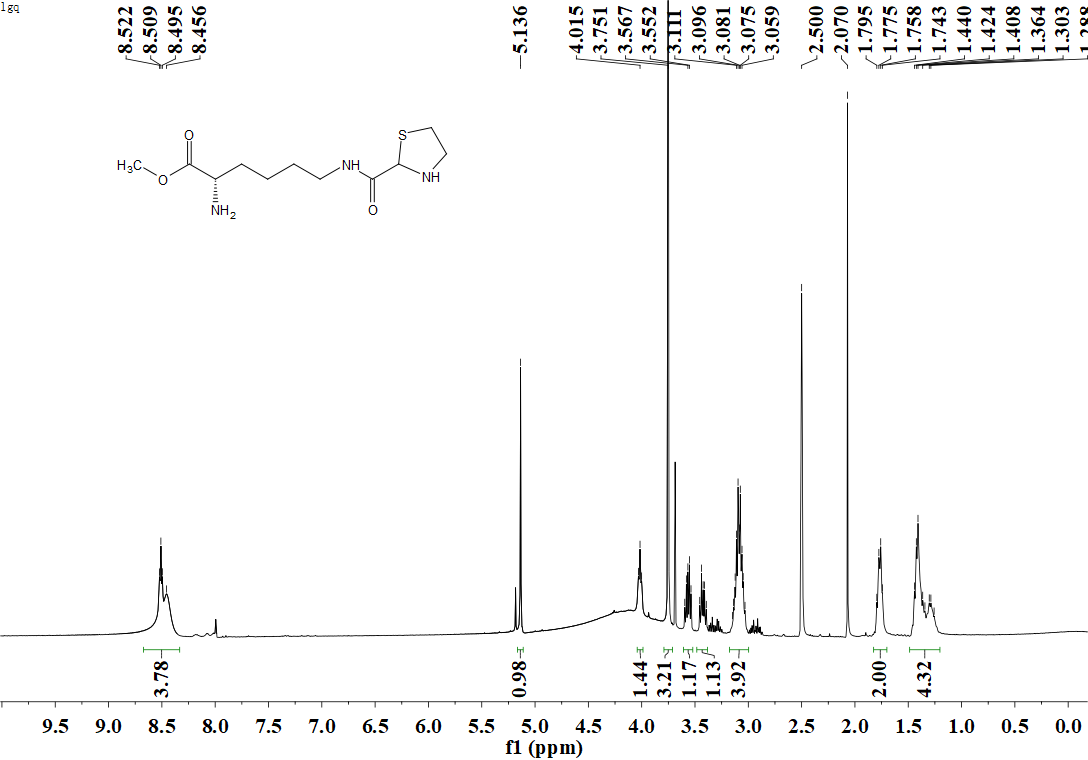


**Figure S17.** ^1^H NMR spectrum of ThzK-OMe (400 MHz, DMSO-*d_6_*).

References

1. X. Bi, K. K. Pasunooti, J. Lescar, C.-F. Liu, *Bioconjugate chem*. **2017**, *28*, 325.
2. H. Zhou, J. W. Cheung, T. Carpenter, S. K. Jones, N. H. Luong, N. C. Tran, S. E. Jacobs, S. A. Galbada Liyanage, T. A. Cropp, J. Yin, *Bioorg Med Chem Lett.* ***2020***, *30*, 126876.
